# Body-Shape Concerns, Eating Regulation, and Mediterranean Diet Adherence in a Romanian Convenience Sample: A Cross-Sectional Self-Determination Theory Perspective

**DOI:** 10.3390/bs16091690

**Published:** 2026-09-19

**Authors:** Cristina Georgescu, Cristina Filip, Ada Moldovan, Florina Daniela Ruta

**Affiliations:** 1 Nutrition and Dietetics Program, Faculty of Pharmacy, George Emil Palade University of Medicine, Pharmacy, Science and Technology of Târgu Mureș, 540142 Târgu Mureș, Romania; adaognean@gmail.com; 2 Department of Biochemistry and Environmental Chemistry, Faculty of Pharmacy, George Emil Palade University of Medicine, Pharmacy, Science and Technology of Târgu Mureș, 540139 Târgu Mureș, Romania; 3 Department of Community Nutrition and Food Safety, Faculty of Medicine, George Emil Palade University of Medicine, Pharmacy, Science and Technology of Târgu Mureș, 540139 Târgu Mureș, Romania; florina.ruta@umfst.ro

**Keywords:** body image, body shape concerns, self-determination theory, autonomous motivation, controlled motivation, eating regulation, Mediterranean diet, MEDAS, adults, Romania

## Abstract

Autonomous motivation may support healthy eating, whereas body-shape concerns may fuel controlled eating regulation; the two had not been examined jointly with Mediterranean diet adherence in Romanian adults. In this cross-sectional study, a convenience sample of 224 predominantly female (87.1%), university-educated Romanian adults completed the Body Shape Questionnaire-16B (BSQ-16B), the Regulation of Eating Behaviors Scale (REBS), and a Romanian-adapted 14-item Mediterranean Diet Adherence Screener (MEDAS). The theory-derived expectations were not preregistered. Fit of the six-factor REBS structure improved substantially when cross-loadings between adjacent regulations were allowed, though discriminant validity remained limited; a bifactor analysis supported the BSQ-16B total score. BSQ-16B scores were associated with introjected (β = 0.65) and controlled regulation (β = 0.54) but not with autonomous styles or adherence (ρ = −0.03). Autonomous motivation was associated with adherence after adjustment (b = 0.63 points per standard deviation; 95% confidence interval 0.31–0.94), as was physical activity; in an exploratory subscale model, integrated regulation showed the strongest unique association. Body-shape concerns were coupled with internal pressure but showed little evidence of an association with MEDAS-assessed adherence, whereas autonomous regulation emerged as a relevant correlate. The findings are consistent with, but do not test, autonomy-supportive counseling.

## 1. Introduction

The Mediterranean dietary pattern (Willett et al., 1995) is among the best-documented approaches for the prevention of cardiovascular disease and other chronic conditions (Dinu et al., 2018; Estruch et al., 2018). Despite this evidence, adherence has been declining worldwide, including within Mediterranean regions (da Silva et al., 2009; Vilarnau et al., 2019), and tends to be low in Central and Eastern European countries: in a nationwide Slovenian survey, the mean score on the 14-item Mediterranean Diet Adherence Screener (MEDAS) was only 5.6 out of 14 (Poklar Vatovec et al., 2023). Research on the determinants of adherence has concentrated on sociodemographic factors such as education, income, and age (Mendonça et al., 2022; Poklar Vatovec et al., 2023), including within dietary intervention trials (Downer et al., 2016), and, to a lesser extent, on psychosocial factors (Ruggiero et al., 2019; Tsofliou et al., 2022). A recent systematic review of 37 studies from Mediterranean countries concluded that socio-cognitive and interpersonal determinants of adherence remain underexplored and should be studied within a broader socio-ecological context (C. Obeid et al., 2025). Understanding which psychological characteristics accompany better adherence is a prerequisite for designing counseling approaches that support durable dietary change.

Self-determination theory (SDT) offers a well-established framework for this question (Deci & Ryan, 2000; Ryan & Deci, 2000). SDT distinguishes qualitatively different regulation styles arranged along a continuum of internalization: intrinsic, integrated, and identified regulation constitute autonomous motivation, in which behavior is experienced as self-endorsed and congruent with personal values; introjected and external regulation constitute controlled motivation, in which behavior is driven by internal pressure (guilt, shame, contingent self-worth) or external contingencies; amotivation denotes the absence of intentional regulation. Applied to eating, the Regulation of Eating Behaviors Scale (REBS) operationalizes these six styles (Pelletier et al., 2004). Reviews and meta-analyses across health behaviors consistently indicate that autonomous regulation is associated with more stable behavioral engagement, whereas controlled regulation shows weak or negative associations (Ng et al., 2012; P. J. Teixeira et al., 2012; Verstuyf et al., 2012), and relative autonomy has been shown to translate into intentional dietary behavior (Hagger et al., 2006).

For overall diet quality, the largest test to date comes from the PREDISE study of 1097 Canadian adults, in which self-determined motivation measured with the REBS was the psychosocial variable most strongly associated with a national healthy-eating index (Carbonneau et al., 2021). For the Mediterranean diet specifically, the evidence has grown rapidly and points to multiple interacting psychosocial processes. In 1940 Italian adults, perceived behavioral control was the strongest antecedent of the intention to adhere, followed by anticipated emotions and subjective norms, with health and mood motives acting only indirectly and past adherence moderating all paths (Carfora et al., 2022). In a randomized controlled trial with 726 Italian adults, a manipulation fostering autonomous relative to controlled motivation acted on intentions toward the diet, whereas follow-up adherence was most strongly predicted by past adherence (Caso et al., 2024), and a prospective model integrating SDT with the theory of planned behavior found that autonomous motivation related to Mediterranean-diet behavior primarily through intention (Canova et al., 2026). Most directly relevant, a 2026 study of 365 Italian young adults related the SDT regulation styles, assessed with a shortened REBS, to adherence measured with a 15-item questionnaire within an integrated SDT/planned-behavior model: intrinsic motivation was directly associated with adherence, integrated regulation acted indirectly through perceived behavioral control, and identified and introjected regulation showed no effects (Gentile et al., 2026). The relation between the regulatory spectrum and Mediterranean-diet behavior is therefore no longer unexplored. What remains missing is an examination of the six REBS regulation styles in relation to MEDAS-assessed adherence in an adult, non-Mediterranean Eastern European sample, and, above all, their joint examination with body image.

Body image is a second psychological candidate. Body-shape concern, the core preoccupation captured by the Body Shape Questionnaire (BSQ) and its short forms (Cooper et al., 1987; Evans & Dolan, 1993), is often assumed to motivate dietary improvement. SDT-based research suggests a more specific picture: body dissatisfaction has been linked to controlled, particularly introjected, regulation of eating and to dysfunctional rather than healthy eating patterns (Pelletier & Dion, 2007; Pelletier et al., 2004), and, in a sample of 208 women, body dissatisfaction was associated with more controlled regulation of eating whereas global self-determination protected against internalization of the thin ideal (Kopp & Zimmer-Gembeck, 2011). Studies relating body image to Mediterranean diet adherence have largely treated body dissatisfaction as an outcome or a correlate of weight status rather than modeling it together with motivational quality (Ruggiero et al., 2019; Tsofliou et al., 2022). Whether body-shape concern is associated with adherence to a healthy dietary pattern once motivational quality is considered therefore remains an open question.

The present study addressed these gaps in a sample of Romanian adults, a population for which, to our knowledge, no adult data on psychological correlates of MEDAS-assessed adherence have been published; the available Romanian evidence concerns children and adolescents (Pira et al., 2021). Its contribution is the joint examination of body-shape concern, the full REBS regulatory spectrum, and MEDAS-assessed adherence, contrasting body-image-related internal pressure with autonomous eating regulation. Drawing on SDT, we examined three expectations: (H1) autonomous motivation for eating regulation is positively associated with Mediterranean diet adherence; (H2) controlled motivation is not positively associated with adherence; and (H3) greater body-shape concern is associated with higher controlled motivation. As a secondary aim, we examined which of the six REBS regulation styles are associated with adherence when modeled simultaneously. These expectations were derived from SDT but were not preregistered, and they were formalized after data collection (see Section 2.3); the study should therefore be read as exploratory and hypothesis-generating rather than as a confirmatory test. Because the design was cross-sectional, all expectations concerned associations rather than causal or temporal effects.

## 2. Materials and Methods

### 2.1. Study Design and Participants

This observational cross-sectional study used a structured, self-administered online questionnaire distributed in Romanian through Google Forms (Google LLC, Mountain View, CA, USA) and is reported in accordance with the Strengthening the Reporting of Observational Studies in Epidemiology (STROBE) guidance for cross-sectional studies (von Elm et al., 2008). The study protocol was approved by the Research Ethics Committee of the George Emil Palade University of Medicine, Pharmacy, Science and Technology of Târgu Mureș (decision no. 3949 of 5 February 2026), and the study was conducted in accordance with the Declaration of Helsinki. Adults were recruited by convenience sampling between 22 February and 30 April 2026: the survey link was shared on the first author’s Instagram and Facebook pages and through WhatsApp, including WhatsApp and Facebook groups, and was passed on by recipients (snowball dissemination); no paid advertising, institutional mailing list, or patient group was used, and the link was openly accessible to anyone who received it. Eligibility criteria were age ≥ 18 years, knowledge of Romanian, provision of informed consent by continuing after the study-information page, and submission of the complete questionnaire; all items were mandatory, so no item-level data were missing. The form required sign-in to a Google account and was set to accept one response per account, which limited, though it could not entirely prevent, repeated participation; e-mail addresses were not collected, and names and Internet Protocol (IP) addresses were not recorded, so the only automatically stored variable was the submission timestamp, which was used solely for the duplicate audit and is not part of the analytic dataset; responses were therefore anonymous to the research team, in the sense that no direct identifiers were collected or accessible to the researchers, although participation required authentication with the platform. Responses were stored in a password-protected account accessible only to the first author. No CAPTCHA, cookie-based, or IP-based restriction was applied, and no attention-check items or completion-time measures were available. Response integrity was therefore examined after collection (Section 3.1): the exported dataset comprised 225 submissions, and a response-level audit identified one exact duplicate (two submissions received 7 s apart that were identical across all 54 scale items and all sociodemographic variables), whose later copy was removed as a probable double submission, leaving *N* = 224 unique records for the primary analyses; additional screens for near-duplicate response vectors, invariant (straight-lined) responding, and temporally clustered submissions are reported in Section 3.1. The platform did not record the number of people who viewed but did not submit the form, so a response rate could not be calculated; the denominator is unknown, and the sample cannot be considered representative of Romanian adults. Because the survey was presented as a study about eating and body image, self-selection related to interest in nutrition is likely. The study used the fixed sample accrued during the collection window; no effect-size-based a priori sample-size calculation was performed, and inference therefore emphasizes 95% confidence intervals and the precision of the estimates. Preliminary bivariate results based on an interim sample of the first 160 respondents were presented at the Marisiensis International Congress and published as a conference abstract (Georgescu & Ruța, 2026); the present article reports the complete dataset and the full covariate-adjusted analyses, which supersede those preliminary estimates. The relation between that interim analysis and the present hypotheses is described in Section 2.3.

### 2.2. Measures

Body-shape concern over the previous four weeks was assessed with the 16-item Body Shape Questionnaire, alternate form B (BSQ-16B) (Cooper et al., 1987; Evans & Dolan, 1993). Items are rated from 1 (never) to 6 (always); the total score ranges from 16 to 96, with higher scores indicating greater concern. In the present sample, internal consistency was excellent (Cronbach’s α = 0.955; McDonald’s ω = 0.956).

Motivation for the regulation of eating behaviors was assessed with the 24-item REBS (Pelletier et al., 2004), rated from 1 (does not correspond at all) to 7 (corresponds exactly). Six four-item subscale scores (intrinsic, integrated, identified, introjected, external regulation, and amotivation) were computed as item means, together with the standard composites of autonomous motivation (mean of the intrinsic, integrated, and identified items) and controlled motivation (mean of the introjected and external items). Internal consistency was good to excellent for the autonomous composite (α = 0.94; ω = 0.939) and its subscales (α = 0.869–0.891), the controlled composite (α = 0.80; ω = 0.816), introjected regulation (α = 0.724), and external regulation (α = 0.781), but only modest for the four-item amotivation subscale (α = 0.615; ω = 0.653; corrected item–total correlations 0.31–0.51), whose coefficients are therefore interpreted cautiously throughout. The ω coefficients are one-factor ω-total values (McDonald, 1999); for the two composites, which SDT conceptualizes as combining distinguishable regulations, ω is reported as an internal-consistency index of the unit-weighted composite score and not as evidence of unidimensionality, and a model-based ω-total derived from the six-factor confirmatory model (Section 2.3) is reported as a check. Full descriptive statistics and reliability coefficients for all scales and subscales are given in Supplementary Table S1.

Adherence to the Mediterranean diet was assessed with a Romanian adaptation of the 14-item MEDAS developed within the PREDIMED program (Martínez-González et al., 2012; Schröder et al., 2011), referred to hereafter as the Romanian-adapted MEDAS. Each criterion met scores one point (total 0–14); items phrased as consumption limits were worded so that an affirmative answer indicated meeting the criterion. For descriptive purposes and for the ordinal sensitivity model, scores were also categorized as low (0–5), moderate (6–9), and high (≥10) adherence; these are the conventional cutoffs of the original MEDAS (Martínez-González et al., 2012), and they have not been validated for the adapted Romanian version. The MEDAS has been applied and evaluated across Mediterranean and non-Mediterranean European populations (García-Conesa et al., 2020). The adaptation retained the 14 criteria of the original instrument and their frequency thresholds, but did not reproduce all of its serving definitions: a quantitative serving size was specified for vegetables (≥200 g raw or ≥150 g cooked) and wine (one glass = 100–150 mL), the fruit item included whole fruit, juice, and separately consumed portions without a quantitative serving size, the gram-based serving definitions of the original for meat, butter/margarine/cream, legumes, fish, and nuts were not displayed, the original’s requirement that at least one daily vegetable serving be raw or as salad was not included, and the white-meat criterion referred to chicken and turkey versus red meat. Locally typical foods were named as examples for processed meat, legumes, and sweetened beverages, and the “sofrito” criterion, for which no Romanian term exists, was rendered descriptively as a sauce of tomato, garlic, onion, or leek in olive oil. These simplifications may have made some criteria less strict than in the original; the outcome is therefore described throughout as Romanian-adapted MEDAS or MEDAS-assessed adherence, and its scores and categories should be compared with other MEDAS studies with caution (Section 4.6). The full Romanian wording, with the scoring rules, is given in Materials S4. Because the MEDAS is a formative index of heterogeneous dietary behaviors rather than a reflective scale, internal consistency is not an appropriate psychometric criterion and is not reported.

Sociodemographic and behavioral characteristics recorded were age, sex, self-reported height and weight (from which body mass index, BMI, was computed), educational level, history of past dieting, whether the respondent was currently following a specific eating pattern (no; for weight loss; for weight maintenance; for medical reasons), current weight goal (trying to lose weight; to maintain weight; to gain weight; no specific goal), habitual physical activity frequency (rarely or never; 1–2, 3–4, or ≥5 times/week), and the frequency of eating in situations of stress or strong emotion (never to very often, five levels). Socioeconomic status or income, food insecurity, urban versus rural residence, current nutrition counseling, smoking, alcohol use beyond the MEDAS wine item, and chronic disease status were not collected. The primary adjustment covariates were age, sex, BMI, education, and physical activity; the remaining behavioral characteristics were used in sensitivity analyses (Section 2.3). In the regression models, sex was coded 1 = female (male = reference); educational attainment was entered as a three-level ordinal variable (non-tertiary education [high school or post-secondary non-tertiary], university, and postgraduate education); physical activity was entered as a four-level ordinal frequency score; and continuous predictors were standardized.

The three instruments were administered in Romanian versions prepared for this study through forward translation by the first author (C.G., who holds a bachelor’s degree in nutrition and dietetics and a master’s degree in clinical and community nutrition, is a native Romanian speaker, and was familiar with the SDT terminology of the REBS from her graduate research), followed by a bilingual linguistic review, item by item against the source instruments, by A.M., a co-author who holds the same two degrees and is also a qualified teacher of English, with discrepancies resolved by consensus, and a small comprehensibility pretest with four Romanian-speaking adults from the target population, who completed the online questionnaire and were asked to flag any item they found unclear; no comprehension problems were reported and no wording was modified. Because the instrument was not modified after the pretest, the four pretest responses, which were submitted through the same online form and are the first four records received (before the survey link was disseminated), were retained in the primary analysis; they are flagged in Supplementary Data S1, and their exclusion is examined as a sensitivity analysis (Section 3.6). The procedure was informed by selected steps of the World Health Organization process for the translation and adaptation of instruments (World Health Organization, 2016) but did not include independent back-translation or a multidisciplinary expert panel, and no formal psychometric validation of the Romanian versions had been undertaken before this study; the factor structure of the BSQ-16B and REBS was therefore examined in the present sample (Section 2.3). The Romanian wording of the MEDAS, together with the administration instructions, response anchors, and scoring rules of all three instruments, is provided in Materials S4; the Romanian item wordings of the BSQ-16B and REBS are available from the authors on request, subject to the permission of the respective copyright holders.

### 2.3. Statistical Analysis

**Transparency statement.** The research question, the three instruments, and the sociodemographic and behavioral variables to be collected were fixed before data collection, but no preregistration or written statistical analysis plan existed. The directional expectations H1–H3 and the hierarchical regression strategy were formalized after data collection was complete and after the interim bivariate results on the first 160 respondents (Georgescu & Ruța, 2026) had been examined. All analyses reported here should therefore be regarded as exploratory; the labels H1–H3 are retained for readability and do not imply prospective hypothesis testing.

Continuous variables are summarized as mean (standard deviation, SD) and median; categorical variables as *n* (%). Because the principal scores departed from normality (Shapiro–Wilk *p* < 0.05), bivariate associations used Spearman’s ρ, with 95% confidence intervals (CIs) for key coefficients obtained by bootstrap (5000 resamples). The family of nine bivariate associations with the MEDAS (six subscales, two composites, and the BSQ-16B) was controlled for multiplicity with the Benjamini–Hochberg false-discovery-rate procedure, and both *p-* and q-values are reported (Benjamini & Hochberg, 1995). Internal consistency was quantified with Cronbach’s α and one-factor McDonald’s ω (McDonald, 1999).

**Factor structure of the Romanian instruments.** Because the Romanian versions had not been validated, their dimensionality was examined with confirmatory factor analysis (CFA) by maximum likelihood, treating the 6- and 7-point items as continuous, which is acceptable for ordinal indicators with five or more categories (Rhemtulla et al., 2012); because the items were not multivariate normal, the Satorra–Bentler mean-scaled χ^2^ and the corresponding scaled fit indices were computed, and nested models were compared with the scaled difference test (Satorra & Bentler, 1994, 2001). For the REBS, the hypothesized six-factor independent-clusters model was compared with one-factor, three-factor (autonomous, controlled, and amotivation items loading directly on three factors), and second-order (autonomous and controlled higher-order factors) alternatives. Because independent-clusters CFA fixes all cross-loadings to zero, which is known to depress fit and inflate factor correlations for SDT motivation scales (Marsh et al., 2014; Stenling et al., 2023), an unrestricted six-factor exploratory model (equivalent in fit to an exploratory structural equation model, ESEM) with oblique target rotation toward the hypothesized pattern was also fitted. As reported in Section 3.2, this model was retained as the best-fitting representation of REBS dimensionality in the present sample because it provided the best fit while recovering the intended pattern. For the BSQ-16B, which is scored as a total, a one-factor confirmatory model, one-, two-, and three-factor exploratory models and, because the exploratory solutions indicated two content facets within a strong general factor, a correlated two-facet model and a bifactor model (a general factor plus two orthogonal specific factors) were fitted; the bifactor indices ω hierarchical (ωH), explained common variance (ECV), and H (Reise, 2012; Rodriguez et al., 2016) served to evaluate whether a sufficiently dominant general factor supported use of the total score. Fit was evaluated with the comparative fit index (CFI), the Tucker–Lewis index (TLI), the root mean square error of approximation (RMSEA) with its 90% CI, and the standardized root mean square residual (SRMR), interpreted in context rather than as decision rules, because their values depend on the number of indicators, on measurement quality, and on the estimator (Marsh et al., 2004; McNeish et al., 2018; Xia & Yang, 2019), with the stricter benchmarks proposed by Hu and Bentler (1999) (CFI/TLI ≈ 0.95, RMSEA ≈ 0.06, SRMR ≈ 0.08) and the more lenient conventional values (CFI/TLI ≈ 0.90; RMSEA ≤ 0.08; Browne & Cudeck, 1993) used as reference points. Because the items are ordered categories, the same models were also estimated with the weighted least squares mean- and variance-adjusted (WLSMV) estimator for ordinal indicators in lavaan 0.7-2 (Rosseel, 2012) in R 4.6.1 (R Core Team, Vienna, Austria), with scaled-and-shifted difference tests (Satorra, 2000). Because the scaled CFI, TLI, and RMSEA under WLSMV are not comparable with the maximum-likelihood reference values (Xia & Yang, 2019; Savalei, 2021), the robust versions of Savalei (2021) are also reported, and SRMR, which is comparatively robust to the estimation method (Shi & Maydeu-Olivares, 2020) and has been specifically evaluated for ordinal factor analysis (Shi et al., 2020), was considered alongside RMSEA for the ordinal models (Supplementary Table S13). Estimation details, standardized loadings, factor correlations, factor-based ω coefficients, residual correlations, the rotated exploratory solutions, the bifactor solution, and a polychoric unweighted least squares (ULS) cross-check are given in Supplementary Tables S2–S4 and S10–S14. Because a CFA in the same sample used for the substantive analyses does not constitute independent validation, these analyses are labeled exploratory/internal and are interpreted as evidence about dimensionality, not as a validation study.

**Primary model and covariate rationale.** The primary analysis was a hierarchical linear regression on the continuous MEDAS score: step 1 entered age, sex, BMI, education, and physical activity; step 2 added the BSQ-16B; step 3 added the autonomous, controlled, and amotivation composites. The covariate set was selected on theoretical grounds, before the reported multivariable models were fitted, as the sociodemographic and lifestyle characteristics most consistently associated with Mediterranean diet adherence in previous work (Mendonça et al., 2022; Poklar Vatovec et al., 2023; Ruggiero et al., 2019) and plausibly related to motivational quality: age, sex, and education were treated as exogenous characteristics that may shape body-shape concern, motivational quality, and adherence, and physical activity and BMI as concurrent lifestyle and anthropometric characteristics that may be common correlates of body-shape concern (BMI in particular), motivation, and adherence, although BMI could also lie on a pathway from diet to body-shape concern. Past dieting was not included in the primary model because its causal position is ambiguous (it may be a common cause of current body-shape concern and dietary behavior, but equally a consequence of body-shape concern, and hence a mediator of any BSQ-16B–adherence association, or of earlier motivational quality) and was examined in sensitivity analyses rather than assumed to be a confounder; current weight goal, current adherence to a specific eating pattern, and emotional-eating frequency were treated in the same way. Because socioeconomic status, food insecurity, residence, nutrition counseling, smoking, alcohol use, and chronic disease were not measured, “adjusted” and “independently associated” refer throughout to adjustment for the measured covariates only, and residual confounding cannot be excluded. The order of blocks is descriptive and does not imply temporal or causal precedence; the coefficients of all predictors at each step, and the alternative order in which the motivational composites are entered before the BSQ-16B, are reported in Supplementary Table S6. Linear regression on the 0–14 score was retained as the primary model because the score is a count of criteria met whose distribution was approximately symmetric and unimodal, well away from the bounds (mean 7.2, SD 2.0), because coefficients are interpretable directly in MEDAS points, and because the residual diagnostics (Section 3.4) were satisfactory; because robust standard errors address heteroskedasticity but not the bounded, discrete nature of the outcome, ordinal and binomial models were added as sensitivity analyses. Coefficients are reported per SD of the standardized predictors with 95% CIs, using HC3 heteroskedasticity-robust standard errors; R^2^ change between steps was tested with the conventional incremental F test and, because that test assumes homoskedasticity, with an HC3-robust joint Wald test of each added block. Model diagnostics comprised residual normality (Shapiro–Wilk), the Breusch–Pagan test, and variance-inflation factors (VIFs). The mirror-image models regressed each regulation style, in turn, on the BSQ-16B with the same covariates; of these eight models, the associations with introjected regulation and the controlled composite correspond to H3, whereas the remaining six were exploratory, and Benjamini–Hochberg q-values are reported across the family of eight. In a secondary analysis, the six REBS subscales were entered simultaneously in place of the composites, retaining all covariates and the BSQ-16B, with Benjamini–Hochberg correction across the six motivational coefficients (Supplementary Tables S7 and S8); the coefficients in this model are interpreted as unique associations conditional on the other regulations, not as evidence that one regulation is intrinsically more important than another. A BSQ-16B × autonomous motivation interaction and a sex × autonomous motivation interaction were each examined in a single supplementary model. Because the H2 statement that controlled motivation is “not positively associated” with adherence cannot be established by a nonsignificant test, and no equivalence framework was prespecified, results bearing on H2 are described as the absence of evidence for an association, with the CI as the measure of what the data can exclude.

**Sensitivity analyses.** The primary model was refitted (a) in the full 225-record dataset including the duplicate; (b) in women only; (c) with a 13-item MEDAS excluding the wine item, whose cultural relevance in Romania is uncertain; (d) after excluding observations exceeding the Cook’s-distance screening threshold of 4/*n* (Cook, 1977), a screening rule and not a judgment that such observations are invalid (all observations, including one participant with BMI 43.0 kg/m^2^, were retained in the primary analysis); (e) after excluding any respondent identified as straight-lining in the response-integrity screen; (f) after excluding the four comprehensibility-pretest respondents (Section 2.2); (g) with past dieting added as a covariate; (h) with education and physical activity entered as categorical indicator variables instead of ordinal linear terms, with joint tests for each set (Supplementary Table S9); (i) with the additionally measured behavioral characteristics (past dieting, current weight-loss attempt, current adherence to a specific eating pattern, and frequency of emotional eating) added simultaneously; (j) as a proportional-odds ordinal logistic regression on the conventional low/moderate/high categories of the original MEDAS applied to the Romanian-adapted score (Section 2.2), with the same predictors, reporting odds ratios (ORs) per SD, a bootstrap CI (2000 resamples) for the autonomous coefficient, and a likelihood-ratio test of the proportional-odds assumption against a generalized ordered logit model; and (k) as a binomial-logit model treating the MEDAS score as the number of criteria met out of 14, with sandwich (robust) standard errors. To check that the findings did not depend on unit-weighted scoring of a translated instrument, the primary, mirror-image, and secondary models were also refitted (l) with CFA-based Bartlett factor scores averaged into the composites, (m) with composites weighted by the standardized CFA loadings, and (n) after excluding the two REBS items with the largest residual correlations in the CFA (the confirmatory model was used for these scorings because its loadings are defined without rotation). Exploratory indirect-effect analyses (percentile bootstrap, 10,000 resamples) and a Harman single-factor diagnostic (Podsakoff et al., 2003), which is a weak descriptive screen and is not treated as evidence that common-method bias is absent, are reported in the Supplementary Materials. All analyses were conducted in Python 3.12.3 (Python Software Foundation, Beaverton, OR, USA; pandas 3.0.2, NumPy 2.4.4, SciPy 1.17.1, matplotlib 3.10.8, openpyxl 3.1.5) with custom NumPy/SciPy implementations of all procedures, except the WLSMV models, which were estimated in R 4.6.1 with lavaan 0.7–2 (Rosseel, 2012); the complete code (with a run-all script and README) and the R output are provided in Supplementary Script S3. Scale totals were recomputed from item-level data, and two-sided *p* < 0.05 or q < 0.05 indicated statistical significance.

## 3. Results

### 3.1. Sample Characteristics and Response Integrity

The sample comprised 224 adults (87.1% women) with a mean age of 40.7 (SD 10.0) years (range 20–73) and a mean BMI of 24.9 (SD 4.8) kg/m^2^ (range 16.4–43.0). Participants were predominantly highly educated, and most reported past dieting. Characteristics are shown in Table 1. The mean Romanian-adapted MEDAS score was 7.2 (SD 2.0); by the conventional cutoffs of the original MEDAS, adherence was low (0–5) in 21.0%, moderate (6–9) in 66.1%, and high (≥10) in 12.9% of participants.

Beyond the exact duplicate described in Section 2.1, the response-integrity screen of the 225 submissions found no other pair sharing 52 or more of the 54 scale-item responses; of the 11 other pairs of submissions received within 60 s of each other, none shared more than 34 of 54 responses (median inter-submission interval 18.7 min), which is consistent with independent respondents rather than repeated entries. One respondent gave the same rating to all 24 REBS items (2 on every item, including both intrinsic and amotivation items), a pattern that is internally inconsistent and was treated as probable straight-lining; this record was retained in the primary analysis, and its exclusion is examined in Section 3.6. Two respondents answered “never” to all 16 BSQ-16B items, which is the scale minimum and a plausible response for adults without body-shape concern; these records were retained.

### 3.2. Reliability and Factor Structure of the Romanian Instruments

Descriptive statistics and reliability coefficients for all scales are shown in Supplementary Table S1; as noted in Section 2.2, all multi-item psychological scales showed good to excellent internal consistency (ω between 0.73 and 0.96) except the four-item amotivation subscale (ω = 0.653). The model-based ω-total derived from the six-factor confirmatory model was 0.949 for the autonomous composite and 0.823 for the controlled composite, close to the one-factor values (0.939 and 0.816). The motivational profile was dominated by autonomous regulation, with identified regulation showing the highest mean, whereas controlled motivation and amotivation were low.

The results of the factor analyses are summarized in Table 2 and detailed in Supplementary Tables S2–S4 and S10–S14. For the REBS, the hypothesized six-factor independent-clusters model fitted far better than the one-factor, three-factor, and second-order alternatives (scaled Δχ^2^ tests, all *p* < 0.001; the second-order model also yielded a boundary solution), which were rejected, but its absolute fit was mixed: under maximum likelihood, scaled RMSEA was within the conventional range (0.068, 90% CI 0.059–0.077), whereas scaled CFI (0.892), TLI (0.874), and SRMR (0.086) fell short of the benchmarks; under WLSMV, scaled CFI (0.952) and TLI (0.944) reached them, whereas scaled RMSEA (0.092, 90% CI 0.084–0.100) and SRMR (0.096) did not, and the robust categorical-data indices were lower still (CFI = 0.787, TLI = 0.752, RMSEA = 0.142, 90% CI 0.130–0.155). All 24 items loaded on their intended factor (standardized loadings 0.39–0.95; median 0.77; the three weakest being two amotivation items and one external-regulation item), and the factor correlations followed the simplex pattern expected along the internalization continuum: the three autonomous factors were strongly interrelated (r = 0.61–0.85), introjected and external regulation and amotivation were moderately to strongly interrelated (r = 0.53–0.71), and correlations between autonomous and controlled factors were small (r from −0.22 to 0.29; Supplementary Table S3). The misfit of the independent-clusters model was concentrated in cross-loadings between adjacent regulations (Supplementary Table S4). When these were freed in the six-factor exploratory (ESEM-equivalent) model, fit was good under maximum likelihood (scaled CFI = 0.985, TLI = 0.972, RMSEA = 0.032, 90% CI 0.010–0.047, SRMR = 0.025), the improvement over the independent-clusters model was significant (scaled Δχ^2^(90) = 286.8, *p* < 0.001), and 19 of 24 items had their largest loading on the intended factor (Supplementary Table S11), the same contrast between confirmatory and exploratory representations reported for the English original (CFI 0.868 versus 0.969; Stenling et al., 2023). The WLSMV estimation, used as an ordinal sensitivity check, reproduced the relative superiority of the exploratory model (scaled-and-shifted Δχ^2^(90) = 175.7, *p* < 0.001; scaled RMSEA = 0.047, 90% CI 0.033–0.059, SRMR = 0.027; robust RMSEA = 0.090, 90% CI 0.066–0.113, CFI = 0.947; 19 of 24 items on the intended factor; Table 2, Supplementary Table S13), but its solution was improper (a negative residual variance for the amotivation item C6, whose uniqueness is at its lower bound under maximum likelihood as well; Supplementary Table S13), so it is treated as a sensitivity analysis rather than as confirmatory evidence and the maximum-likelihood solution is the reference; fixing the uniqueness of C6 at small positive values changed the maximum-likelihood fit only minimally (scaled RMSEA remained 0.032; Table S13). The exploratory six-factor model was therefore retained as the best-fitting representation of REBS dimensionality in the present sample, and the independent-clusters model is reported as the hypothesized structure and the basis of the model comparisons. Under WLSMV, the factor correlations were higher than under maximum likelihood (autonomous factors 0.76–0.86; introjected–external 0.68; external–amotivation 0.78; Supplementary Table S13), consistent with limited discriminant validity between adjacent regulations (a polychoric ULS cross-check agreed; Supplementary Table S10). Taken together, the Romanian REBS recovered the intended six-factor pattern in its main features, but discriminant validity between adjacent regulations, among the three autonomous regulations and between amotivation and external regulation, was limited, as reported for the original and Portuguese versions (Stenling et al., 2023; D. S. Teixeira et al., 2021). This motivated prioritizing the composite-level analyses (the autonomous and controlled composites, whose components were substantially intercorrelated within composites and only weakly across them) and treating the subscale-level analysis as exploratory.

For the BSQ-16B, all 16 items loaded substantially on a common factor (standardized loadings 0.64–0.86 by maximum likelihood, 0.66–0.89 under WLSMV; first eigenvalue 9.7 versus 1.2 for the second; ω = 0.956), but the one-factor model did not fit adequately under maximum likelihood (scaled CFI = 0.847, TLI = 0.824, RMSEA = 0.112 [0.101–0.124]; SRMR = 0.060) or under WLSMV (scaled CFI = 0.963, TLI = 0.957, RMSEA = 0.125 [0.114–0.137]; robust CFI = 0.858, RMSEA = 0.150 [0.135–0.166]; SRMR = 0.063), indicating local dependence beyond the general factor. An exploratory two-factor model fitted acceptably (scaled CFI = 0.953, TLI = 0.937, RMSEA = 0.067 [0.053–0.082], SRMR = 0.033), separating items about fear of fatness and preoccupation with shape (B1–B4, B10) from items about avoidance, body checking, and feeling large (factor correlation 0.69; a three-factor model fitted slightly better still; Supplementary Table S12). A bifactor model with a general factor and two orthogonal specific factors for these facets showed substantially improved fit under maximum likelihood (scaled CFI = 0.958, TLI = 0.943, RMSEA = 0.064 [0.049–0.078], SRMR = 0.034) and by the scaled WLSMV indices (CFI = 0.990, TLI = 0.987, RMSEA = 0.070 [0.056–0.084], SRMR = 0.034), whereas its robust categorical-data RMSEA was 0.096 (90% CI 0.076–0.116) with CFI = 0.951 (Table 2), and its indices showed that the general factor dominates (ωH = 0.905, ECV = 0.844, H = 0.959 by maximum likelihood; ωH = 0.933, ECV = 0.867 under WLSMV; Supplementary Table S14). By the criteria of Rodriguez et al. (2016), these indices indicate a dominant general factor; the bifactor model thus provides additional internal support for the use of the total score, which is how the instrument is used here, while confirming local multidimensionality, with the caveat that the facets were identified post hoc in the same sample. The substantive analyses were nevertheless repeated with the two exploratory facets and with model-based scorings of the REBS in Section 3.6.

### 3.3. Body-Shape Concerns and Eating Regulation

**Figure 1:**
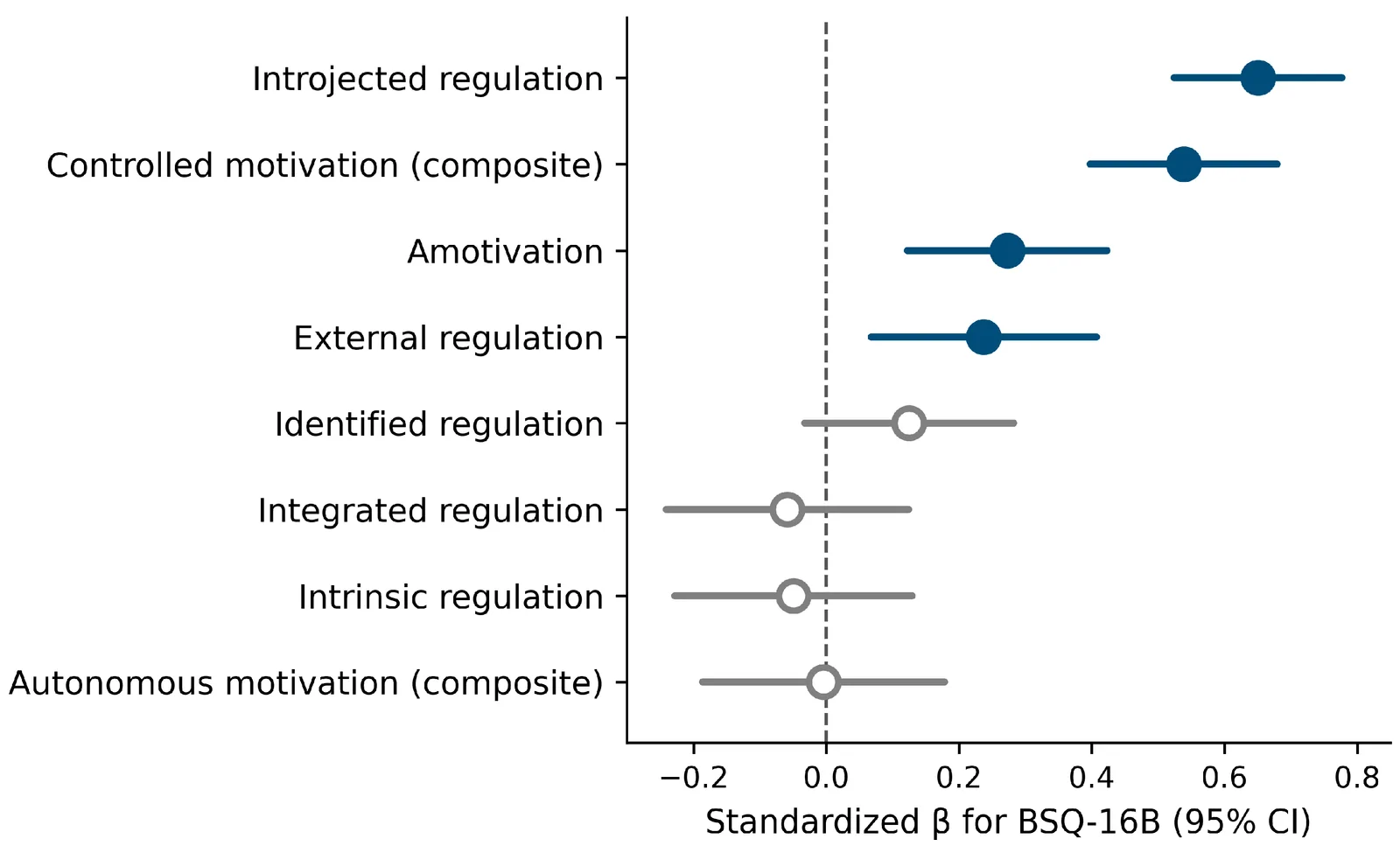
Adjusted standardized associations (β with 95% confidence intervals, CI) between body-shape concerns (Body Shape Questionnaire-16B, BSQ-16B) and the six eating-regulation styles and two composites of the Regulation of Eating Behaviors Scale (REBS). Models are adjusted for age, sex, body mass index, education, and physical activity (HC3-robust standard errors). Blue markers indicate intervals excluding zero.

In covariate-adjusted models (Table 3, Figure 1), higher BSQ-16B scores were strongly associated with introjected regulation (β = 0.650 per SD; 95% CI 0.524–0.777; *p* < 0.001; q < 0.001) and with the controlled motivation composite (β = 0.539; 95% CI 0.398–0.680; *p* < 0.001; q < 0.001), consistent with the theory-derived expectation (H3), and more modestly with external regulation and amotivation (exploratory; q = 0.013 and 0.001). In contrast, no association was observed between the BSQ-16B and any of the autonomous regulation styles (intrinsic, integrated, or identified) or the autonomous composite, for which the adjusted coefficient was close to zero with a narrow interval (β = −0.004; 95% CI −0.186 to 0.178). The bivariate association between the BSQ-16B and introjected regulation was ρ = 0.60 (95% CI 0.50–0.69); it was present in both sexes (women ρ = 0.57, 95% CI 0.45–0.66; men ρ = 0.49, 95% CI 0.14–0.75, *n* = 29).

### 3.4. Correlates of Mediterranean Diet Adherence

Bivariately, the MEDAS score correlated positively with integrated (ρ = 0.43; 95% CI 0.32–0.54), intrinsic (ρ = 0.31), and identified regulation (ρ = 0.29), and with the autonomous composite (ρ = 0.40; 95% CI 0.28–0.50; all q < 0.001), and negatively with amotivation (ρ = −0.26; q < 0.001) and external regulation (ρ = −0.15; q = 0.035). No association with adherence was observed for introjected regulation (ρ = −0.003; q = 0.96), the controlled composite (ρ = −0.08; q = 0.27), or, notably, the BSQ-16B (ρ = −0.03; q = 0.80; 95% CI −0.16 to 0.11).

**Figure 2:**
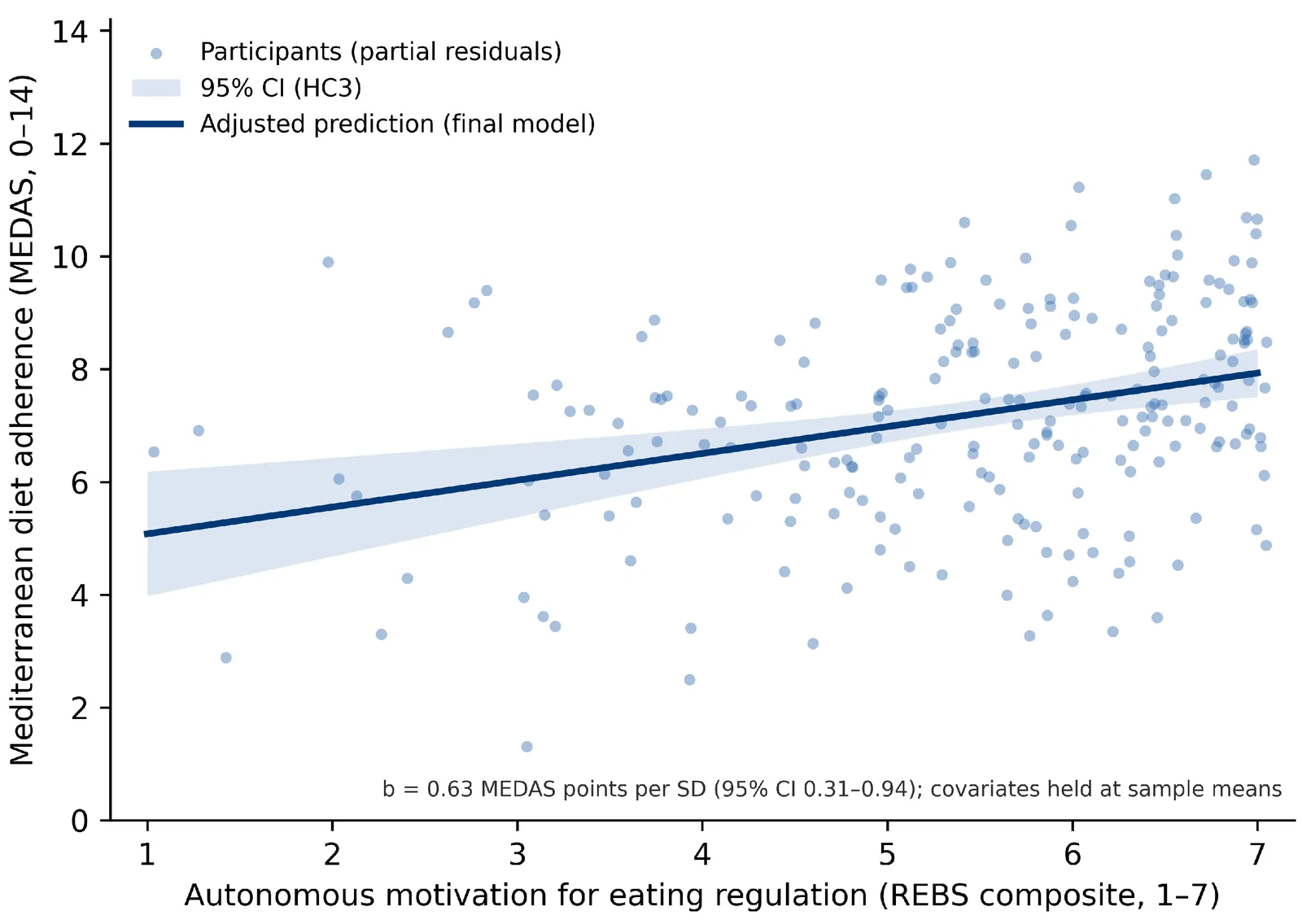
Adjusted association between autonomous motivation for eating regulation (Regulation of Eating Behaviors Scale (REBS) composite, 1–7) and Mediterranean diet adherence (Mediterranean Diet Adherence Screener (MEDAS), 0–14). The line shows the prediction from the final hierarchical model (Table 4, step 3) across the observed range of autonomous motivation, with the remaining predictors (age, sex, body mass index, education, physical activity, BSQ-16B, controlled motivation, and amotivation) held at their sample means; the band is the 95% confidence interval (CI) based on HC3-robust standard errors. Points are partial residuals (component-plus-residual values) for the 224 participants, jittered horizontally for visibility.

The hierarchical regression is summarized in Table 4 (final step) and Supplementary Table S6 (all steps). Covariates explained R^2^ = 0.102, with physical activity as the only covariate associated with adherence. Adding the BSQ-16B left the model essentially unchanged (b = 0.03 MEDAS points per SD at this step; 95% CI −0.26 to 0.31; ΔR^2^ = 0.0001; incremental F *p* = 0.86; HC3-robust Wald χ^2^(1) = 0.03, *p* = 0.85). Adding the motivational composites improved the model substantially (ΔR^2^ = 0.095; F(3,214) = 8.47; *p* < 0.001; HC3-robust Wald χ^2^(3) = 22.3, *p* < 0.001): autonomous motivation was associated with adherence after adjustment for the measured covariates (b = 0.63 MEDAS points per SD; 95% CI 0.31–0.94; *p* < 0.001; Figure 2), consistent with the theory-derived expectation (H1), whereas for controlled motivation no evidence of a positive association was observed (b = −0.09; 95% CI −0.42 to 0.24; *p* = 0.60); this does not establish the absence of a meaningful association, although the interval excludes positive associations larger than about a quarter of a MEDAS point per SD. No evidence of an association was observed for amotivation (b = −0.23; 95% CI −0.54 to 0.08; *p* = 0.14). Physical activity remained associated with adherence in the final model (b = 0.38 per level; 95% CI 0.10–0.67; *p* = 0.009). When the block order was reversed, the motivational composites entered after the covariates added ΔR^2^ = 0.093 (*p* < 0.001), and the BSQ-16B entered last added ΔR^2^ = 0.003 (*p* = 0.40), so the descriptive conclusion did not depend on the order of entry. Residuals were normally distributed (Shapiro–Wilk *p* = 0.38) and homoscedastic (Breusch–Pagan *p* = 0.33), and all variance-inflation factors were below 2 (maximum 1.82). Neither the BSQ-16B × autonomous motivation interaction (*p* = 0.53) nor the sex × autonomous motivation interaction (b = 0.17; 95% CI −0.64 to 0.98; *p* = 0.67) was significant, but the sex-specific estimates differed markedly in precision: the bivariate association between autonomous motivation and adherence was ρ = 0.39 (95% CI 0.26–0.50) in the 195 women and ρ = 0.31 (95% CI −0.11 to 0.64) in the 29 men.

### 3.5. Secondary Analysis of the Six Regulation Styles

The six subscales were substantially intercorrelated, especially within the autonomous group (Spearman ρ: intrinsic–integrated 0.70, integrated–identified 0.73, intrinsic–identified 0.63; introjected–external 0.45; external–amotivation 0.46; Supplementary Table S8). When they were entered simultaneously in place of the composites, retaining all covariates and the BSQ-16B (Supplementary Table S7), only integrated regulation showed a unique association with adherence (b = 1.11 MEDAS points per SD; 95% CI 0.61–1.61; Benjamini–Hochberg q = 0.0001 across the six motivational coefficients; all other q ≥ 0.23; model R^2^ = 0.252; maximum VIF = 3.6). This coefficient represents the component of integrated regulation that is not shared with intrinsic and identified regulation and does not establish that integrated regulation is intrinsically more important than the other autonomous forms; given the intercorrelations among the autonomous subscales, the result is reported as secondary and interpreted as hypothesis-generating. The same pattern was obtained with CFA-based Bartlett factor scores in place of the subscale means (integrated regulation b = 1.11; 95% CI 0.62–1.59; q < 0.001; all other q ≥ 0.20).

### 3.6. Sensitivity and Supplementary Analyses

The association between autonomous motivation and adherence was essentially unchanged across all sensitivity analyses (Table 5): in the full 225-record dataset including the duplicate, among women only, with the 13-item MEDAS excluding the wine item, after excluding the 17 observations exceeding the Cook’s-distance screening threshold (maximum D = 0.052), after excluding the one respondent with invariant REBS responses, after excluding the four comprehensibility-pretest respondents (*n* = 220), with past dieting added as a covariate (past dieting b = 0.02; 95% CI −0.64 to 0.68), with education and physical activity entered as categorical indicators (neither the education indicators, joint *p* = 0.87, nor a linear misspecification of physical activity, joint *p* = 0.055, affected the motivational estimates; Supplementary Table S9), and with past dieting, current weight-loss attempt, current adherence to a specific eating pattern, and emotional-eating frequency added simultaneously (none of the added variables was associated with adherence, all *p* ≥ 0.14). The BSQ-16B was not associated with adherence in any of these models (all *p* ≥ 0.25; ΔR^2^ ≤ 0.0001 when added to the covariate model), including the model without the pretest respondents (b = 0.10; 95% CI −0.25 to 0.45), in which its adjusted associations with introjected (β = 0.64), controlled (β = 0.53), and autonomous regulation (β = 0.00) were also unchanged. In the proportional-odds model of the conventional low/moderate/high categories, autonomous motivation was associated with a higher adherence category (odds ratio per SD = 1.56; 95% CI 1.13–2.15; bootstrap 95% CI 1.14–2.28), as was physical activity (OR = 1.69; 1.20–2.38), whereas the BSQ-16B (OR = 1.24; 0.85–1.80), controlled motivation, and amotivation were not, and the proportional-odds assumption was not rejected (χ^2^(9) = 5.83; *p* = 0.76); the binomial-logit model gave the same picture (Table 5; BSQ-16B OR = 1.04; 0.95–1.14). The results did not depend on unit-weighted scoring of the translated REBS: with Bartlett factor scores from the six-factor CFA averaged into the composites, with loading-weighted composites, and after excluding items C5 and C12, the autonomous-motivation coefficient ranged from 0.58 to 0.63 (Table 5), and in the corresponding mirror-image models the BSQ-16B remained strongly associated with introjected regulation (β = 0.66; *p* < 0.001) and unassociated with the autonomous composite (β = 0.01; *p* = 0.95). When the two BSQ-16B facets identified in Section 3.2 replaced the total score, neither facet alone was associated with adherence (b = 0.25, 95% CI −0.09 to 0.60, and b = −0.10, 95% CI −0.44 to 0.23), both were associated with introjected regulation (β = 0.37 and 0.33), and the autonomous-motivation coefficient was unchanged (b = 0.64); when the two facets were entered together (r = 0.80), their coefficients diverged in sign, a difference-score pattern reported for transparency (Supplementary Table S12) but not interpreted, given the post hoc derivation and the collinearity of the facets. The stability of the results across these alternative scorings reduces, but does not eliminate, the concern that they depend on the measurement properties of the translated instruments. The exploratory indirect-effect analyses (Supplementary Table S5) provided no statistical evidence of an indirect association between body-shape concern and adherence through the autonomous regulations, introjected or external regulation, or the two composites; the only exception was a small negative indirect association through amotivation (−0.09 MEDAS points per SD of the BSQ-16B; 95% CI −0.17 to −0.01), which is not interpreted causally. The Harman-style single-factor diagnostic is reported in Supplementary Materials, Section S2 (first unrotated component: 21% of the variance of the 54 items).

## 4. Discussion

### 4.1. Main Findings

In this predominantly female, highly educated convenience sample of Romanian adults, higher body-shape concern was strongly associated with introjected and overall controlled regulation of eating, showed no association with the autonomous regulations, and showed little evidence of an association with MEDAS-assessed Mediterranean diet adherence (ΔR^2^ < 0.001 when added to the covariate model). Among the motivational variables examined, autonomous motivation was the only construct associated with adherence after adjustment, about 0.63 MEDAS points per SD, and physical activity was the only covariate associated with adherence; in an exploratory mutually adjusted subscale model, integrated regulation showed the strongest unique association. The Romanian REBS recovered the intended six-factor pattern in its main features, with limited discriminant validity between adjacent regulations, and the substantive pattern was stable across covariate, model-form, and scoring sensitivity analyses. The following sections take each of these findings in turn, placing it against previous research before interpreting it.

### 4.2. Autonomous Motivation as One Correlate of Mediterranean Diet Adherence

Reviews and meta-analyses across health behaviors consistently report that autonomous regulation accompanies more stable behavioral engagement, whereas controlled regulation shows weak or negative associations (Ng et al., 2012; P. J. Teixeira et al., 2012; Verstuyf et al., 2012), and prospective evidence links autonomous motivation to long-term weight-related outcomes (Silva et al., 2011). For diet quality specifically, the largest test to date is the PREDISE study, in which REBS-assessed self-determined motivation was the psychosocial variable most strongly associated with a national healthy-eating index in 1097 Canadian adults (Carbonneau et al., 2021). The present association between autonomous motivation and MEDAS-assessed adherence is consistent with that body of work and with the most recent Mediterranean-diet study to use the REBS: among 365 Italian young adults, intrinsic motivation was directly associated with adherence and integrated regulation acted through perceived behavioral control, whereas identified and introjected regulation showed no effects (Gentile et al., 2026). Our secondary analysis, in which integrated regulation carried the composite association, differs from that study, in which intrinsic motivation showed the direct association, but the two studies also differ in population, adherence instrument (a 15-item frequency questionnaire versus the MEDAS), REBS version, and, most importantly, in the inclusion of socio-cognitive mediators; both converge on the autonomous end of the continuum. Three further points deserve comment. First, the magnitude (roughly two-thirds of a MEDAS point per SD, and an odds ratio of 1.6 per SD for a higher adherence category) was modest in absolute terms and comparable to the contribution of physical activity, the only sociodemographic or lifestyle covariate associated with adherence in these data. That physical activity remained associated with adherence after adjustment for the measured covariates is itself noteworthy: healthy lifestyle behaviors commonly cluster, and autonomous motivation should be read as one relevant correlate alongside such behaviors, not as the sole correlate identified. Second, the broader literature indicates that Mediterranean-diet adherence is shaped by several psychosocial processes that this study did not measure. Perceived behavioral control, anticipated emotions, subjective norms, and past adherence were the strongest antecedents of intention in 1940 Italian adults, with motives acting only indirectly (Carfora et al., 2022); intention and past behavior carried much of the association between autonomous motivation and behavior in prospective and experimental work (Canova et al., 2026; Caso et al., 2024); and a recent systematic review concluded that socio-cognitive and interpersonal determinants remain underexplored relative to socio-economic ones (C. Obeid et al., 2025). Autonomous motivation is therefore best presented as one important psychological correlate among several plausible determinants rather than as the uniquely relevant psychological characteristic. Third, the subscale-level result converges with recent evidence that the integrated component of eating regulation is the one most consistently related to healthier food habits beyond global self-determination in a bifactor analysis of the REBS in 1447 middle-aged women (Stenling et al., 2023) and with person-centered analyses in which profiles marked by self-determined eating regulation showed the most adaptive eating outcomes (Martin et al., 2024). Our cross-sectional association is compatible with several readings that the design cannot distinguish: autonomous or integrated regulation may precede adherence, possibly through intention; existing adherence may become progressively internalized over time; or a third characteristic such as health consciousness or nutrition knowledge may contribute to both.

### 4.3. Body-Shape Concerns and Controlled Regulation of Eating

The strong association between body-shape concern and introjected regulation replicates, in an adult community sample, the link between body dissatisfaction and pressured, self-worth-contingent eating regulation described by Pelletier and Dion (2007), documented again in 208 women in whom body dissatisfaction was associated with more controlled regulation of eating (Kopp & Zimmer-Gembeck, 2011), and found once more among 939 exercisers, in whom body-image-satisfied profiles reported the highest autonomous and lowest controlled motivation for eating (Salvador et al., 2026). The present result therefore replicates and extends an established pattern to a Romanian community sample rather than establishing a new phenomenon. Within SDT, introjection is a partial internalization: the person pressures herself with guilt, shame, and contingent self-worth, but the behavior is not integrated with abiding values (Deci & Ryan, 2000; Ryan & Deci, 2000). The literature on psychologically driven eating supports the same coupling from a different angle. Body dissatisfaction rises in tandem with emotional eating, routine restraint, and lower intuitive eating in young women (Eck et al., 2022); body-image concerns are associated with unhealthy weight-control behaviors across adolescence and emerging adulthood (Neumark-Sztainer et al., 2006), and eating-disorder risk and emotional eating covary in university students (Arslan & Alataş, 2023). In our sample, the frequency of eating in situations of stress or strong emotion was measured and, when added to the model, neither altered the motivational estimates nor was itself associated with adherence, which suggests that the body-shape–introjection link is not simply a proxy for emotional eating. The exploratory indirect-effect analyses provided no statistical evidence of an indirect association between body-shape concern and adherence through the autonomous regulations, introjected or external regulation, or the composites under the specified cross-sectional model, and only a small negative indirect association through amotivation; because such models do not establish temporal order, these results cannot be interpreted causally and do not demonstrate the presence or absence of an indirect psychological process.

### 4.4. Body-Shape Concerns and MEDAS-Assessed Adherence: Little Evidence of an Association

Body-shape concern is often assumed to motivate dietary improvement, yet the evidence relating body image to diet quality, whether or not the Mediterranean pattern is the outcome, is mixed and depends on what is measured. In a five-year study of adolescents, lower body satisfaction did not predict healthier behaviors and predicted more unhealthy weight-control practices (Neumark-Sztainer et al., 2006); among Polish adolescents, weight dissatisfaction was related to some unhealthy dietary behaviors but not to others (Wawrzyniak et al., 2020); in a Swiss national nutrition survey, women dissatisfied with their weight had lower diet quality, an association largely explained by BMI (Chatelan & Carrard, 2021); and among 261 young American women, body dissatisfaction was strongly related to maladaptive eating styles but not to fruit and vegetable, fat, or sugar-sweetened beverage intake (Eck et al., 2022). Studies relating body image specifically to Mediterranean diet adherence have largely treated body dissatisfaction as an outcome or a correlate of weight status rather than modeling it together with motivational quality (Ruggiero et al., 2019; Tsofliou et al., 2022). Our results align most closely with Eck et al. (2022): body-shape concern traveled with internal pressure and with controlled regulation, but showed little evidence of an association with MEDAS-assessed adherence, and this held after adjustment for BMI, past dieting, and current weight goals. Two qualifications are essential. First, a nonsignificant coefficient does not demonstrate the absence of an association; what the data show is that any linear association between the BSQ-16B and the MEDAS in this sample is likely to be small (adjusted b = 0.14 MEDAS points per SD; 95% CI −0.21 to 0.49; bivariate ρ 95% CI −0.16 to 0.11), the same conclusion was reached under ordinal and binomial modeling of the outcome, and neither of the two facets of the BSQ-16B identified in the exploratory factor analysis was associated with adherence on its own. Second, the MEDAS captures one specific form of dietary behavior, conformity to a Mediterranean pattern, and does not capture restrictive eating, dieting, food avoidance, compensatory behaviors, or apparently health-oriented food choices with which body dissatisfaction is known to be associated. The finding is therefore specific to MEDAS-assessed adherence and should not be read as showing that body-shape concern has no behavioral expression. A plausible reading, consistent with SDT, is that internally pressured regulation is enacted inconsistently and does not translate into a coherent Mediterranean-pattern diet, whereas value-congruent, identity-level regulation does; adaptive eating styles such as intuitive eating, which are inversely related to body dissatisfaction, have themselves been associated with higher MEDAS scores in a large sample of Turkish young adults (Ongun Yilmaz et al., 2026). Longitudinal and experimental designs are needed to test these interpretations.

### 4.5. Adherence Levels and Sample Composition in Context

The mean Romanian-adapted MEDAS score of 7.2 was higher than the 5.6 reported in a nationwide Slovenian survey (Poklar Vatovec et al., 2023), but such cross-country comparisons should be interpreted cautiously: the Romanian adaptation simplified some serving definitions (Section 2.2), so part of any difference may reflect item operationalization rather than dietary behavior, and the low/moderate/high categories used descriptively here are the conventional cutoffs of the original MEDAS, not thresholds validated for this adaptation. With that caveat, both values remain far from the high-adherence range, consistent with the generally modest adherence documented outside, and increasingly inside, the Mediterranean basin (C. A. Obeid et al., 2022; Vilarnau et al., 2019). Comparison with other MEDAS studies in non-classical Mediterranean settings is informative mainly for what it says about sample composition. Among 729 Turkish university students, women scored 7.2 ± 2.0, and men 6.5 ± 2.2, and sociodemographic and anthropometric variables explained about one-fifth of the variance in MEDAS scores (Alataş et al., 2026); the women’s mean coincides with ours, although the two implementations of the instrument are not strictly comparable, and our sample was 87% female, mostly university-educated, and self-selected into a nutrition-themed survey. The same composition matters for the motivational variables: three-quarters of participants reported past dieting, and the means of the autonomous and especially the identified regulations were high, so the sample is likely to over-represent adults already interested in healthy eating, and the motivational distribution may be restricted or displaced relative to the general population. The observed levels of adherence and motivation, and the strength of the associations, therefore cannot be extrapolated to Romanian adults in general, among whom adherence varies with sociodemographic characteristics that this sample does not represent (Mendonça et al., 2022; Poklar Vatovec et al., 2023).

### 4.6. Strengths and Limitations

Strengths include theory-driven expectations, three established instruments spanning body image, motivational quality, and dietary pattern, an internal examination of the factor structure of the translated instruments, multiplicity control, robust-variance estimation with full model diagnostics, and convergent sensitivity analyses across covariate sets, model forms, and scoring methods; the key null associations were estimated with reasonably narrow confidence intervals. Several limitations must temper interpretation. First, the cross-sectional design precludes causal or directional inference, and the findings concern correlates rather than determinants of adherence. Second, the hypotheses and the analysis plan were not preregistered and were formalized after data collection and after an interim bivariate analysis; although the expectations follow directly from SDT and the analyses are standard, the study is exploratory, and its results require confirmation in an independent, preferably preregistered, sample. Third, the study was an open online survey with an unknown denominator, with only the one-response-per-account setting and a post hoc integrity screen as safeguards against repeated participation, and with probable self-selection related to interest in nutrition, which may itself be associated with the principal variables (interest in healthy eating, autonomous regulation, body concerns, and past dieting); the convenience sample (predominantly female, highly educated, and with extensive dieting experience) therefore limits generalizability in more than a generic sense (Section 4.5). Fourth, only 29 men participated. The women-only sensitivity analysis shows that the principal association is not driven by the male subgroup, but it does not resolve the sex imbalance: the sex-specific estimates for men are highly uncertain, associations between body-image discrepancy and eating motivation have been reported to differ by sex (Salvador et al., 2026), and the present data cannot establish that the same motivational pattern occurs to the same degree in Romanian men; measurement invariance across sex could not be tested. Fifth, all measures were self-reported and collected in a single session from the same source, so common-method bias cannot be excluded; the Harman diagnostic is only a weak descriptive screen and is not evidence that such bias is absent, although the different response formats across instruments and the central null association argue against a purely method-driven pattern. Sixth, the Romanian versions of the instruments were prepared for this study without independent back-translation or an expert panel, were pretested in only four adults (whose retention in the analytic sample did not affect the results; Section 3.6), and have not undergone independent psychometric validation. The factor analyses reported here were conducted in the same sample used for the substantive analyses, are therefore internal evidence about dimensionality rather than validation, and gave mixed results (Section 3.2): the six-factor REBS structure was clearly superior to the alternatives under both estimators, but the independent-clusters model did not reach the conventional benchmarks on all indices (its RMSEA exceeded 0.08 under WLSMV), substantially improved fit was obtained in the exploratory six-factor model allowing cross-loadings between adjacent regulations, which was retained as the best-fitting representation of its dimensionality in the present sample (RMSEA 0.032 by maximum likelihood, 0.047 by the scaled and 0.090 by the robust WLSMV computation), and discriminant validity between adjacent regulations was limited, as also reported for other versions of the instrument and for independent-clusters models of SDT scales more generally (Section 3.2); the one-factor BSQ-16B model did not fit, and the use of the total score is supported by a bifactor model (RMSEA 0.064, 0.070, and 0.096 by the same three computations) whose general factor accounted for 84–87% of the common variance (ωH = 0.90–0.93), which also confirmed local multidimensionality, with facets identified post hoc in the same sample. Consequently, the subscale-level result is exploratory; the composite-level results are the ones on which the conclusions rest, and the stability of the substantive results across model-based scorings reduces but does not eliminate measurement-related uncertainty; an independent validation sample and tests of measurement invariance across sex and other groups are needed before the Romanian versions can be considered validated. The Romanian-adapted MEDAS omitted some serving definitions and the requirement of at least one raw vegetable serving (Section 2.2); these simplifications may have made some criteria less strict, limited the comparability of scores with other MEDAS studies, and were not tested beyond the wine-item sensitivity analysis. The low/moderate/high categories are the conventional cutoffs of the original MEDAS, not thresholds validated for the Romanian adaptation, so they were used only descriptively and in the ordinal sensitivity model. Seventh, the amotivation subscale showed modest reliability, and its factor is weakly determined in the exploratory solution (the uniqueness of one item, C6, lies at its boundary; Section 3.2), so its coefficients are interpreted cautiously. Eighth, past dieting, current weight goals, and emotional eating were measured and examined only in sensitivity analyses because their causal position is ambiguous, and socioeconomic status, food insecurity, urban versus rural residence, current nutrition counseling, smoking, alcohol use beyond the wine item, and chronic disease status were not collected; each could plausibly relate to both motivational quality and adherence, so residual confounding cannot be excluded, “adjusted” associations refer to the measured covariate set only, and the study did not measure the socio-cognitive determinants (perceived behavioral control, intention, anticipated emotions, norms) that recent work identifies as important. Finally, MEDAS adherence and anthropometrics were self-reported, and the wine item may have uncertain cultural relevance in Romania, although its exclusion left the results unchanged.

### 4.7. Practical Implications

The implications of these data for nutrition counseling are hypotheses suggested by the observed associations, not conclusions supported by an intervention test, because the study did not manipulate counseling strategies, measure changes in motivation or adherence over time, or establish temporal order; moreover, experimental and longitudinal Mediterranean-diet research suggests that autonomous motivation acts partly through intention and other socio-cognitive processes rather than directly determining subsequent behavior (Canova et al., 2026; Caso et al., 2024). With those caveats, the findings are consistent with the rationale for autonomy-supportive counseling described in the SDT literature (P. J. Teixeira et al., 2012; Verstuyf et al., 2012): the motivational profile that accompanied better MEDAS-assessed adherence in these data was one in which eating was connected to personally endorsed values and identity, whereas the profile that accompanied body-shape concern (internal pressure, guilt, and contingent self-worth) had no counterpart in this measure of dietary pattern. The data therefore caution against assuming that intensifying body-focused concern will translate into a healthier dietary pattern, but whether counseling that reduces such concern or fosters integration of eating with personal values improves adherence can only be established by longitudinal and experimental studies.

## 5. Conclusions

In a predominantly female, highly educated convenience sample of Romanian adults, body-shape concerns were strongly associated with controlled, especially introjected, regulation of eating, whereas little evidence of an association with MEDAS-assessed Mediterranean diet adherence was observed after adjustment for the measured covariates; autonomous eating regulation emerged as one relevant psychological correlate of adherence after adjustment for measured covariates, alongside physical activity, and this pattern was robust across model forms and scorings, whereas the finding that integrated regulation showed the strongest unique association among the six regulation styles remains exploratory. These cross-sectional, non-preregistered findings identify the internalization of eating regulation as a correlate, not a determinant, of a Mediterranean-pattern diet in this sample, and they are consistent with, but do not test, the rationale for autonomy-supportive dietary counseling.

## Figures and Tables

**Table 1:** Sociodemographic and behavioral characteristics of the sample (*N* = 224).

Characteristic	Category	*n*	%
Sex	Women	195	87.1
	Men	29	12.9
Educational level	High school	30	13.4
	Post-secondary (non-tertiary)	7	3.1
	University	122	54.5
	Postgraduate	65	29.0
BMI category	Underweight (<18.5)	10	4.5
	Normal weight (18.5–24.9)	125	55.8
	Overweight (25.0–29.9)	51	22.8
	Obesity (≥30.0)	38	17.0
Past dieting	Yes	163	72.8
	No	61	27.2
Physical activity	Rarely or never	88	39.3
	1–2 times/week	66	29.5
	3–4 times/week	58	25.9
	≥5 times/week	12	5.4

BMI, body mass index.

**Table 2:** Fit of the factor models for the Romanian REBS and BSQ-16B under maximum-likelihood (Satorra–Bentler-scaled) and ordinal (WLSMV) estimation (*N* = 224). The best-fitting representation is listed first for each instrument; the other rows are the hypothesized independent-clusters models and the rejected alternatives, fitted for model comparison.

*(A) Maximum-likelihood estimation, Satorra–Bentler-scaled (Python, Script S3)*							
**Model**	**χ^2^ (df)**	**Scaled χ^2^**	**CFI**	**TLI**	**RMSEA**	**90% CI**	**SRMR**
*REBS*							
Six factors, exploratory (ESEM-equivalent)—best-fitting	226.1 (147)	180.5	0.985	0.972	0.032	0.010–0.047	0.025
Six correlated factors, independent clusters (hypothesized; basis of the difference tests)	629.2 (237)	480.0	0.892	0.874	0.068	0.059–0.077	0.086
Second-order (autonomous, controlled)—rejected	669.9 (244)	513.2	0.880	0.864	0.070	0.062–0.079	0.104
Three factors—rejected	978.0 (249)	724.4	0.788	0.765	0.093	0.085–0.100	0.106
One factor—rejected	1609.5 (252)	1197.7	0.578	0.538	0.130	0.122–0.137	0.162
*BSQ-16B*							
Bifactor: general factor + two orthogonal specific facets—best-fitting	198.4 (88)	167.7	0.958	0.943	0.064	0.049–0.078	0.034
Two facets, exploratory	213.5 (89)	178.9	0.953	0.937	0.067	0.053–0.082	0.033
Two correlated facets, independent clusters	332.5 (103)	283.3	0.906	0.891	0.089	0.076–0.101	0.053
One factor (hypothesized)—inadequate	468.9 (104)	397.0	0.847	0.824	0.112	0.101–0.124	0.060
*(B) Ordinal (WLSMV) estimation, R/lavaan 0.7–2*							
**Model**	**Scaled χ^2^ (df)**	**Scaled CFI/TLI**	**Scaled RMSEA [90% CI]**		**Robust CFI/TLI**	**Robust RMSEA [90% CI]**	**SRMR**
*REBS*							
Six factors, ESEM—ordinal sensitivity analysis ^a^	218.2 (147)	0.992/0.986	0.047 [0.033–0.059]		0.947/0.900	0.090 [0.066–0.113]	0.027
Six correlated factors, independent clusters (hypothesized)	684.6 (237)	0.952/0.944	0.092 [0.084–0.100]		0.787/0.752	0.142 [0.130–0.155]	0.096
Three factors—rejected	1042.5 (249)	0.915/0.906	0.120 [0.112–0.127]		0.712/0.680	0.162 [0.150–0.173]	0.130
One factor—rejected	2021.7 (252)	0.811/0.793	0.177 [0.170–0.185]		0.482/0.433	0.215 [0.205–0.226]	0.203
*BSQ-16B*							
Bifactor: general + two specific facets—best-fitting	184.3 (88)	0.990/0.987	0.070 [0.056–0.084]		0.951/0.933	0.096 [0.076–0.116]	0.034
Two correlated facets, independent clusters	331.1 (103)	0.977/0.973	0.100 [0.088–0.112]		0.906/0.891	0.123 [0.106–0.139]	0.050
One factor (hypothesized)—inadequate	468.3 (104)	0.963/0.957	0.125 [0.114–0.137]		0.858/0.836	0.150 [0.135–0.166]	0.063

CFI, TLI, and RMSEA (with its 90% CI) in Panel A are the Satorra–Bentler-scaled values (conventional values in Supplementary Table S2); the scaled χ^2^ column reports the mean-scaled χ^2^. Exploratory models have all loadings free (echelon identification); their fit does not depend on rotation. Scaled difference tests against the six-factor independent-clusters REBS model: exploratory six-factor model, Δχ^2^(90) = 286.8; three factors, Δχ^2^(12) = 164.0; one factor, Δχ^2^(15) = 525.2; second-order, Δχ^2^(7) = 36.2; all *p* < 0.001. The BSQ-16B models are compared descriptively by their fit and bifactor indices (Table S14); no difference tests are reported because the one-factor model is a boundary case of both alternatives. In Panel B, the scaled and the robust (Savalei, 2021) indices are not on the same scale and are not judged by the same reference values (Section 2.3; Table S13); scaled-and-shifted difference tests (Satorra, 2000) against the six-factor independent-clusters model: three factors Δχ^2^(12) = 231.8, one factor Δχ^2^(15) = 315.7, exploratory six-factor model Δχ^2^(90) = 175.7, all *p* < 0.001. ^a^ Improper solution (negative residual variance for item C6; Table S13); reported as an ordinal sensitivity analysis, not as confirmatory evidence. Standardized loadings, factor correlations, residual correlations, the polychoric ULS cross-check, the rotated exploratory solutions, the WLSMV solutions, and the bifactor solution are given in Supplementary Tables S2–S4 and S10–S14. REBS, Regulation of Eating Behaviors Scale; BSQ-16B, Body Shape Questionnaire-16B; ESEM, exploratory structural equation modeling; WLSMV, weighted least squares mean- and variance-adjusted; CFI, comparative fit index; TLI, Tucker–Lewis index; RMSEA, root mean square error of approximation; SRMR, standardized root mean square residual; CI, confidence interval. Bold type marks the column headings of each panel; italic type marks the panel titles and the instrument subheadings (REBS and BSQ-16B).

**Table 3:** Adjusted associations between body-shape concerns (BSQ-16B) and eating-regulation styles (*N* = 224).

Motivational Outcome	β per SD	95% CI	*p*	q
Introjected regulation ^1^	0.650	0.524, 0.777	<0.001	<0.001
Controlled motivation (composite) ^1^	0.539	0.398, 0.680	<0.001	<0.001
Amotivation	0.273	0.123, 0.423	<0.001	0.001
External regulation	0.237	0.068, 0.407	0.006	0.013
Identified regulation	0.125	−0.032, 0.282	0.118	0.19
Integrated regulation	−0.058	−0.241, 0.124	0.53	0.67
Intrinsic regulation	−0.049	−0.228, 0.129	0.59	0.67
Autonomous motivation (composite)	−0.004	−0.186, 0.178	0.97	0.97

Each row is a separate linear regression of the standardized motivational outcome on the standardized BSQ-16B score, adjusted for age, sex, body mass index, education, and physical activity, with HC3-robust standard errors. BSQ-16B, Body Shape Questionnaire-16B; SD, standard deviation; β, standardized coefficient; CI, confidence interval; q, Benjamini–Hochberg false-discovery-rate-adjusted *p*-value across the eight models. ^1^ Associations corresponding to the theory-derived expectation (H3); the remaining six were exploratory.

**Table 4:** Hierarchical linear regression of Mediterranean diet adherence (MEDAS, 0–14) on covariates, body-shape concerns, and motivational quality (*N* = 224).

Predictor (Step 3)	b	95% CI	*p*
Age	−0.11	−0.37, 0.16	0.43
Sex (female)	−0.27 ^1^	−1.11, 0.57	0.52
Body mass index	0.03	−0.29, 0.35	0.85
Education	0.06 ^2^	−0.39, 0.51	0.79
Physical activity	0.38 ^2^	0.10, 0.67	0.009
BSQ-16B	0.14	−0.21, 0.49	0.43
Autonomous motivation	0.63	0.31, 0.94	<0.001
Controlled motivation	−0.09	−0.42, 0.24	0.60
Amotivation	−0.23	−0.54, 0.08	0.14

Step 1 (covariates): R^2^ = 0.102. Step 2 (+BSQ-16B): ΔR^2^ = 0.0001, *p* = 0.86. Step 3 (+motivational composites): ΔR^2^ = 0.095, F(3,214) = 8.47, *p* < 0.001; final model R^2^ = 0.197. Coefficients b are unstandardized MEDAS-point effects with HC3-robust 95% confidence intervals and *p*-values; continuous predictors are standardized, so their coefficients are per SD. ^1^ Per category (male = reference). ^2^ Per level of the ordinal variable.

**Table 5:** Sensitivity analyses for the association between autonomous motivation and Mediterranean diet adherence (*N* = 224 unless stated).

Model	b per SD (95% CI)	*p*
Primary model (Table 4)	0.63 (0.31, 0.94)	<0.001
Full dataset including duplicate (*N* = 225)	0.63 (0.31, 0.94)	<0.001
Women only (*n* = 195)	0.64 (0.32, 0.96)	<0.001
13-item MEDAS (wine item excluded)	0.64 (0.32, 0.96)	<0.001
Excluding 17 observations with Cook’s D > 4/*n* (*n* = 207)	0.72 (0.46, 0.98)	<0.001
Excluding one respondent with invariant REBS responses (*n* = 223)	0.67 (0.37, 0.97)	<0.001
Excluding the four comprehensibility-pretest respondents (*n* = 220)	0.64 (0.32, 0.95)	<0.001
+Past dieting	0.63 (0.31, 0.95)	<0.001
Education and physical activity as indicator variables	0.62 (0.29, 0.94)	<0.001
+Past dieting, weight-loss attempt, current eating pattern, emotional eating	0.62 (0.30, 0.94)	<0.001
Bartlett factor-score composites (six-factor CFA)	0.58 (0.26, 0.89)	<0.001
Loading-weighted composites	0.61 (0.29, 0.93)	<0.001
Excluding REBS items C5 and C12	0.63 (0.32, 0.94)	<0.001
Ordinal logistic regression, conventional original-MEDAS categories applied to the Romanian-adapted score (OR per SD)	1.56 (1.13, 2.15)	0.006
Binomial-logit model, criteria met out of 14 (OR per SD)	1.20 (1.10, 1.31)	<0.001

All models include age, sex, body mass index, education, physical activity, the BSQ-16B, controlled motivation, and amotivation; linear models use HC3-robust standard errors. b, unstandardized MEDAS points per SD of autonomous motivation; MEDAS, Mediterranean Diet Adherence Screener; SD, standard deviation; BSQ-16B, Body Shape Questionnaire-16B; REBS, Regulation of Eating Behaviors Scale; CFA, confirmatory factor analysis; OR, odds ratio; CI, confidence interval. The BSQ-16B was not associated with adherence in any model (all *p* ≥ 0.25).

## Data Availability

The de-identified item-level dataset (*N* = 225 records, with the removed duplicate and the four pretest respondents flagged; submission timestamps and raw height and weight removed) is provided as Supplementary Data S1 together with its codebook, as it was with the original submission, now including the three additional variables used in the sensitivity analyses; participants were informed that their responses, which were anonymous to the research team, would be used exclusively for academic and scientific research purposes, and the dataset contains no direct identifiers. The complete analysis code (Python, with a separate R/lavaan script for the WLSMV confirmatory analyses) is provided as Supplementary Script S3; together, these reproduce all tables, figures, and analyses that can be derived from Data S1, whereas the timing-based response-integrity checks (Section 3.1) require the timestamped raw export, which is not shared for data-minimization reasons. In addition, the Romanian-adapted MEDAS, with the instructions, response anchors, and scoring rules of all three instruments, is provided as Materials S4 (the Romanian item wordings of the BSQ-16B and REBS are available from the authors on request, subject to the permission of the respective copyright holders).

## Supplementary Materials

The following supporting information can be downloaded at https://www.mdpi.com/article/10.3390/bs16091690/s1: Table S1: Descriptive statistics and reliability of the study scales (*N* = 224); Table S2: Confirmatory factor models of the Romanian REBS and BSQ-16B: conventional (unscaled) and Satorra–Bentler scaled fit indices, and standardized factor loadings (*N* = 224; maximum likelihood); Table S3: Factor correlations in the six-factor REBS model; Table S4: Largest standardized residual correlations (|r| > 0.20 for the REBS; > 0.15 for the BSQ-16B); Table S5: Exploratory indirect-effect analyses: body-shape concern (BSQ-16B, per SD) → regulation style (per SD) → MEDAS adherence, adjusted for age, sex, BMI, education, and physical activity (*N* = 224; percentile bootstrap, 10,000 resamples); Table S6: Coefficients of the hierarchical regression of MEDAS adherence at each step (b, HC3-robust 95% CI, p) and with reversed block order (*N* = 224); Table S7: Complete coefficients of the six-subscale model (MEDAS regressed on covariates, BSQ-16B, and the six REBS subscales entered simultaneously; *N* = 224); Table S8: Spearman correlations among the REBS subscales and composites (*N* = 224); Table S9: Primary model with education and physical activity entered as categorical indicator variables (*N* = 224); Table S10: Polychoric unweighted-least-squares cross-check (custom implementation) compared with the maximum-likelihood solution: standardized loadings; Table S11: Exploratory six-factor model of the Romanian REBS (maximum likelihood; fit equivalent to an exploratory structural equation model): standardized loadings after oblique target rotation toward the hypothesized pattern (target loadings in bold; largest cross-loading and its factor shown), and geomin rotation as a check; Table S12: BSQ-16B: exploratory one-, two-, and three-factor models, the two-factor geomin solution, and facet-level sensitivity analyses of the substantive models; Table S13: Ordinal (WLSMV) confirmatory and exploratory factor models of the Romanian REBS and BSQ-16B estimated in R 4.6.1 with lavaan 0.7–2 (polychoric correlations, diagonally weighted least squares, mean- and variance-adjusted test statistic; *N* = 224), with the scaled and the robust (Savalei, 2021) fit indices; Table S14: Bifactor model of the BSQ-16B (*N* = 224): standardized loadings on the general factor and on the specific facet factor under maximum likelihood (Satorra–Bentler scaling) and under WLSMV, item-level explained common variance (I-ECV), and bifactor indices; Data S1: De-identified item-level dataset (Supplementary_Data_S1_minimal_dataset.xlsx: worksheets Data, Codebook, README; *N* = 225 rows, exact duplicate flagged with inclus_analiza = 0, the four comprehensibility-pretest respondents flagged with pretest = 1) and SPSS scoring syntax (Supplementary_Data_S1_SPSS_syntax.sps); Section S2: Harman single-factor diagnostic; Script S3: Complete analysis code: the Python analysis pipeline (prep.py, stats_utils.py, cfa.py, analysis_original.py, analysis_regression.py, analysis_cfa.py, analysis_scoring.py, analysis_r2.py, analysis_esem.py, analysis_figure2.py, run_all.py, README.md, requirements.txt) together with the R/lavaan script for the WLSMV confirmatory models (lavaan_WLSMV.R) and its output (lavaan_WLSMV_output.txt); Materials S4: Romanian versions of the instruments as administered (Google Forms), with instructions, response options, and scoring rules. The references cited in the Supplementary Materials (Cooper et al., 1987; Evans & Dolan, 1993; Martínez-González et al., 2012; Pelletier et al., 2004; Podsakoff et al., 2003; Satorra, 2000; Savalei, 2021; Schröder et al., 2011; Shi & Maydeu-Olivares, 2020; Xia & Yang, 2019) are all included in the main reference list.
