# Supplementary material for: Body-Shape Concerns, Eating Regulation, and Mediterranean Diet Adherence in a Romanian Convenience Sample: A Cross-Sectional Self-Determination Theory Perspective
Source: Behav Sci (Basel). 2026 Sep 19;16(9):1690. doi: 10.3390/bs16091690 (PMC13603406; doi:10.3390/bs16091690)
Supplement: Supplementary file 1 [file behavsci-16-01690-s001.zip › Supplementary_Materials_R3-edit.pdf]

Supplementary Materials

Body-Shape Concerns, Eating Regulation, and Mediterranean Diet Adherence in a Romanian Convenience Sample: A Cross-Sectional Self-Determination Theory Perspective (Georgescu, Filip, Moldovan, Ruța).

Table S1. Descriptive statistics and reliability of the study scales (N = 224).

Scale (Range)	M (SD)	Median	α	ω
BSQ-16B (16–96)	43.9 (16.7)	41.0	0.955	0.956
MEDAS (0–14)	7.2 (2.0)	7.0	– [1]	– [1]
Autonomous motivation (1–7)	5.43 (1.32)	5.75	0.94	0.939
Intrinsic regulation	5.02 (1.61)	5.25	0.869	0.871
Integrated regulation	5.16 (1.56)	5.50	0.890	0.892
Identified regulation	6.11 (1.25)	6.50	0.891	0.898
Controlled motivation (1–7)	2.87 (1.19)	2.75	0.80	0.816
Introjected regulation	3.65 (1.52)	3.50	0.724	0.725
External regulation	2.09 (1.24)	1.75	0.781	0.825
Amotivation (1–7)	2.26 (1.22)	2.00	0.615	0.653

M, mean; SD, standard deviation; α, Cronbach's alpha; ω, one-factor McDonald's ω (ω-total from a single common factor estimated by unweighted least squares on the Pearson correlation matrix; reproduced by analysis_reliability.py in Script S3). [1] The MEDAS is a formative index of heterogeneous behaviors, for which internal consistency is not an appropriate criterion and is therefore not reported.

Table S2. Confirmatory factor models of the Romanian REBS and BSQ-16B: conventional (unscaled) and Satorra–Bentler scaled fit indices, and standardized factor loadings (N = 224; maximum likelihood).

Model	χ^2 (df)	p	CFI	TLI	RMSEA [90% CI]	SRMR	SB χ^2 (scaling c)	Scaled CFI	Scaled TLI	Scaled RMSEA [90% CI]
REBS, six correlated factors	629.2 (237)	<0.001	0.871	0.850	0.086 [0.078, 0.094]	0.086	480.0 (1.31)	0.892	0.874	0.068 [0.059, 0.077]
REBS, second-order	669.9 (244)	<0.001	0.860	0.842	0.088 [0.081, 0.097]	0.104	513.2 (1.31)	0.880	0.864	0.070 [0.062, 0.079]
REBS, three	978.0 (249)	<0.001	0.761	0.735	0.115 [0.107,	0.106	724.4 (1.35)	0.788	0.765	0.093 [0.085,

Model	χ^2 (df)	p	CFI	TLI	RMSEA [90% CI]	SRMR	SB χ^2 (scaling c)	Scaled CFI	Scaled TLI	Scaled RMSEA [90% CI]
factors					0.122]					0.100]
REBS, one factor	1609.5 (252)	<0.001	0.555	0.513	0.155 [0.148, 0.163]	0.162	1197.7 (1.34)	0.578	0.538	0.130 [0.122, 0.137]
BSQ-16B, one factor	468.9 (104)	<0.001	0.872	0.852	0.125 [0.114, 0.137]	0.060	397.0 (1.18)	0.847	0.824	0.112 [0.101, 0.124]

All models converged. Standardized loadings of the six-factor REBS model and of the one-factor BSQ-16B model:

REBS item	Factor	λ	R^2
C1	amotivation	0.39	0.15
C2	intrinsic	0.82	0.67
C3	external	0.44	0.20
C4	intrinsic	0.81	0.65
C5	identified	0.70	0.50
C6	amotivation	0.40	0.16
C7	introjected	0.66	0.43
C8	introjected	0.64	0.41
C9	external	0.84	0.70
C10	integrated	0.85	0.72
C11	intrinsic	0.74	0.55
C12	introjected	0.58	0.33
C13	external	0.86	0.75
C14	amotivation	0.65	0.43
C15	external	0.77	0.59
C16	identified	0.77	0.59
C17	integrated	0.84	0.70
C18	introjected	0.64	0.41
C19	amotivation	0.74	0.55
C20	integrated	0.77	0.59
C21	integrated	0.82	0.68
C22	identified	0.90	0.80
C23	identified	0.95	0.90
C24	intrinsic	0.80	0.64

BSQ-16B item	λ	R^2
B1	0.76	0.58
B2	0.76	0.58
B3	0.69	0.48
B4	0.84	0.71
B5	0.69	0.47
B6	0.85	0.73
B7	0.64	0.41
B8	0.70	0.50
B9	0.86	0.73
B10	0.77	0.59
B11	0.82	0.67
B12	0.68	0.47
B13	0.85	0.71
B14	0.66	0.44
B15	0.72	0.51
B16	0.84	0.71

Factor-based ω (REBS): intrinsic 0.87, integrated 0.89, identified 0.90, introjected 0.72, external 0.83, amotivation 0.64; BSQ-16B ω = 0.956.

Table S3. Factor correlations in the six-factor REBS model.

	intrinsic	integrated	identified	introjected	external	amotivation
intrinsic	1.00	–	–	–	–	–
integrated	0.85	1.00	–	–	–	–
identified	0.61	0.76	1.00	–	–	–
introjected	0.11	0.14	0.29	1.00	–	–
external	-0.13	-0.17	-0.22	0.53	1.00	–
amotivation	-0.29	-0.35	-0.22	0.58	0.71	1.00

Table S4. Largest standardized residual correlations (|r| > 0.20 for the REBS; > 0.15 for the BSQ-16B).

Model	Item pair	Residual r
REBS six-factor	C5–C11	0.32
REBS six-factor	C11–C12	0.26
REBS six-factor	C5–C17	0.26
REBS six-factor	C3–C12	0.25

Model	Item pair	Residual r
REBS six-factor	C5–C10	0.24
REBS six-factor	C13–C18	0.22
REBS six-factor	C3–C18	0.21
REBS six-factor	C10–C12	0.21
REBS six-factor	C5–C21	0.21
REBS six-factor	C5–C18	-0.21
BSQ-16B one-factor	B1–B2	0.22
BSQ-16B one-factor	B2–B3	0.18
BSQ-16B one-factor	B8–B12	0.18
BSQ-16B one-factor	B7–B14	0.17
BSQ-16B one-factor	B8–B15	0.16

REBS: 59 of 276 item pairs had |r| > 0.10 (maximum 0.32). BSQ-16B: 11 of 120 pairs had |r| > 0.10 (maximum 0.22).

Table S5. Exploratory indirect-effect analyses: body-shape concern (BSQ-16B, per SD) → regulation style (per SD) → MEDAS adherence, adjusted for age, sex, BMI, education, and physical activity (N = 224; percentile bootstrap, 10,000 resamples). Cross-sectional; not interpretable causally.

Regulation style (mediator)	a (BSQ → mediator)	b (mediator → MEDAS)	Indirect effect a × b	95% bootstrap CI	Direct effect c′ (BSQ → MEDAS)
Intrinsic regulation	-0.049	0.461	-0.023	-0.117, 0.053	0.050
Integrated regulation	-0.058	0.771	-0.045	-0.198, 0.081	0.072
Identified regulation	0.125	0.421	0.053	-0.011, 0.128	-0.026
Introjected regulation	0.650	-0.014	-0.009	-0.225, 0.211	0.036
External regulation	0.237	-0.176	-0.042	-0.123, 0.026	0.069
Amotivation	0.273	-0.321	-0.088	-0.166, -0.009	0.114
Autonomous motivation (composite)	-0.004	0.639	-0.002	-0.127, 0.100	0.029
Controlled motivation (composite)	0.539	-0.113	-0.061	-0.227, 0.115	0.088

Coefficients from separate HC3-robust linear models; a and b are per SD; the indirect effect is in MEDAS points per SD of the BSQ-16B. All intervals include zero except for the small negative indirect association through amotivation.

Section S2. Harman single-factor diagnostic. In a Harman-style unrotated principal-components diagnostic of the Pearson correlation matrix of all 54 scale items (16 BSQ-16B, 24 REBS, 14 MEDAS), the first component accounted for 21.0% of the total variance (first eigenvalue 11.34 of 54; 14 eigenvalues greater than 1). Restricted to the 40 psychological items (BSQ-16B and REBS), the first component accounted for 28.2% (7 eigenvalues greater than 1). This is a weak descriptive screen (Podsakoff et al., 2003) and is not evidence that common-method bias is absent.

Script S3. Complete analysis code: the Python analysis pipeline (prep.py, stats_utils.py, cfa.py, analysis_original.py, analysis_regression.py, analysis_cfa.py, analysis_scoring.py, analysis_r2.py, analysis_esem.py, analysis_figure2.py, run_all.py, README.md, requirements.txt) together with the R/lavaan script for the WLSMV confirmatory models (lavaan_WLSMV.R) and its output (lavaan_WLSMV_output.txt). Together, these reproduce all tables, figures, and analyses derivable from Supplementary Data S1 (the timing-based response-integrity checks in Section 3.1 require the timestamped raw export, which is not shared). See README.md for the exact sequence of commands and library versions; the scripts read Supplementary Data S1 directly.

Table S6. Coefficients of the hierarchical regression of MEDAS adherence at each step (b, HC3-robust 95% CI, p) and with reversed block order (N = 224).

Predictor	Step 1	Step 2 (+BSQ-16B)	Step 3 (+motivation)
Age (per SD)	-0.12 (-0.38, 0.15); p = 0.38	-0.11 (-0.38, 0.16); p = 0.42	-0.11 (-0.37, 0.16); p = 0.43
Sex (female)	0.37 (-0.44, 1.19); p = 0.37	0.34 (-0.50, 1.18); p = 0.42	-0.27 (-1.11, 0.57); p = 0.52
Body mass index (per SD)	-0.09 (-0.39, 0.21); p = 0.55	-0.10 (-0.44, 0.23); p = 0.54	0.03 (-0.29, 0.35); p = 0.85
Education (per level)	0.13 (-0.34, 0.60); p = 0.58	0.13 (-0.35, 0.60); p = 0.60	0.06 (-0.39, 0.51); p = 0.79
Physical activity (per level)	0.59 (0.29, 0.88); p < 0.001	0.59 (0.29, 0.88); p < 0.001	0.38 (0.10, 0.67); p = 0.009
BSQ-16B (per SD)	–	0.03 (-0.26, 0.31); p = 0.85	0.14 (-0.21, 0.49); p = 0.43
Autonomous motivation (per SD)	–	–	0.63 (0.31, 0.94); p < 0.001
Controlled motivation (per SD)	–	–	-0.09 (-0.42, 0.24); p = 0.60
Amotivation (per SD)	–	–	-0.23 (-0.54, 0.08); p = 0.14
R^2	0.102	0.102	0.197

Incremental tests: BSQ-16B after covariates $\Delta R^2 = 0.0001$ (F $p = 0.86$; HC3-robust Wald $\chi^2(1) = 0.03$, $p = 0.85$); motivational block after BSQ-16B $\Delta R^2 = 0.095$ ($F(3,214) = 8.47$, $p < 0.001$; HC3-robust Wald $\chi^2(3) = 22.3$, $p < 0.001$). Reversed order: motivational block after covariates $\Delta R^2 = 0.093$ ($p < 0.001$); BSQ-16B entered last $\Delta R^2 = 0.003$ ($p = 0.40$).

Table S7. Complete coefficients of the six-subscale model (MEDAS regressed on covariates, BSQ-16B, and the six REBS subscales entered simultaneously; N = 224).

Predictor	b	95% CI	p	q (BH, six motivational coefficients)
Intercept	6.55	5.36, 7.74	<0.001	–
Age (per SD)	-0.12	-0.37, 0.14	0.36	–
Sex (female)	0.02	-0.77, 0.82	0.95	–
Body mass index (per SD)	0.00	-0.30, 0.31	0.98	–
Education (per level)	-0.02	-0.45, 0.42	0.94	–
Physical activity (per level)	0.33	0.04, 0.62	0.03	–
BSQ-16B (per SD)	0.26	-0.10, 0.63	0.16	–
Intrinsic regulation (per SD)	-0.13	-0.49, 0.23	0.48	0.71
Integrated regulation (per SD)	1.11	0.61, 1.61	<0.001	<0.001
Identified regulation (per SD)	-0.35	-0.74, 0.04	0.08	0.23
Introjected regulation (per SD)	-0.09	-0.44, 0.26	0.61	0.73
External regulation (per SD)	-0.05	-0.36, 0.25	0.73	0.73
Amotivation (per SD)	-0.24	-0.53, 0.06	0.12	0.23

$R^2 = 0.252$; maximum VIF = 3.6. HC3-robust standard errors.

Table S8. Spearman correlations among the REBS subscales and composites (N = 224).

	intrinsic	integrated	identified	introjected	external	amotivation	autonomous	controlled
intrinsic	1.00	–	–	–	–	–	–	–
integrated	0.70	1.00	–	–	–	–	–	–
identified	0.63	0.73	1.00	–	–	–	–	–

	intrinsic	integrated	identified	introjected	external	amotivation	autonomous	controlled
introjected	0.07	0.08	0.14	1.00	–	–	–	–
external	-0.14	-0.21	-0.29	0.45	1.00	–	–	–
amotivation	-0.26	-0.29	-0.35	0.31	0.46	1.00	–	–
autonomous	0.90	0.92	0.82	0.10	-0.23	-0.31	1.00	–
controlled	-0.03	-0.06	-0.04	0.91	0.76	0.41	-0.05	1.00

Table S9. Primary model with education and physical activity entered as categorical indicator variables (N = 224).

Predictor	b	95% CI	p
Age (per SD)	-0.11	-0.38, 0.16	0.42
Sex (female)	-0.23	-1.14, 0.68	0.61
Body mass index (per SD)	0.04	-0.29, 0.37	0.80
Education: university (vs non-tertiary)	-0.05	-0.99, 0.89	0.91
Education: postgraduate (vs non-tertiary)	0.10	-0.90, 1.11	0.84
Physical activity 1–2×/week (vs rarely/never)	0.44	-0.24, 1.12	0.20
Physical activity 3–4×/week (vs rarely/never)	0.94	0.22, 1.66	0.01
Physical activity ≥5×/week (vs rarely/never)	0.81	-0.11, 1.72	0.08
BSQ-16B (per SD)	0.13	-0.24, 0.49	0.49
Autonomous motivation (per SD)	0.62	0.29, 0.94	<0.001
Controlled motivation (per SD)	-0.08	-0.42, 0.26	0.63
Amotivation (per SD)	-0.23	-0.55, 0.09	0.16

R^2 = 0.201; joint test of the education indicators p = 0.87; joint test of the physical-activity indicators p = 0.055.

Table S10. Polychoric unweighted-least-squares cross-check (custom implementation) compared with the maximum-likelihood solution: standardized loadings. The ordinal WLSMV estimation in R/lavaan is reported in Table S13.

Item	Factor	λ (ML)	λ (polychoric ULS)
C1	amotivation	0.39	0.42
C2	intrinsic	0.82	0.81
C3	external	0.44	0.52
C4	intrinsic	0.81	0.78
C5	identified	0.70	0.94

Item	Factor	λ (ML)	λ (polychoric ULS)
C6	amotivation	0.40	0.51
C7	introjected	0.66	0.64
C8	introjected	0.64	0.59
C9	external	0.84	0.89
C10	integrated	0.85	0.89
C11	intrinsic	0.74	0.90
C12	introjected	0.58	0.66
C13	external	0.86	0.90
C14	amotivation	0.65	0.85
C15	external	0.77	0.89
C16	identified	0.77	0.81
C17	integrated	0.84	0.91
C18	introjected	0.64	0.81
C19	amotivation	0.74	0.85
C20	integrated	0.77	0.78
C21	integrated	0.82	0.87
C22	identified	0.90	0.88
C23	identified	0.95	0.89
C24	intrinsic	0.80	0.81
B1	body-shape concern	0.76	0.79
B2	body-shape concern	0.76	0.80
B3	body-shape concern	0.69	0.73
B4	body-shape concern	0.84	0.87
B5	body-shape concern	0.69	0.72
B6	body-shape concern	0.85	0.88
B7	body-shape concern	0.64	0.69
B8	body-shape concern	0.70	0.80
B9	body-shape concern	0.86	0.89
B10	body-shape concern	0.77	0.79
B11	body-shape concern	0.82	0.84
B12	body-shape concern	0.68	0.84
B13	body-shape concern	0.85	0.86
B14	body-shape concern	0.66	0.66
B15	body-shape concern	0.72	0.74
B16	body-shape concern	0.84	0.86

Polychoric factor correlations (REBS): intrinsic–integrated 0.87; intrinsic–identified 0.79; intrinsic–introjected 0.10; intrinsic–external -0.23; intrinsic–amotivation -0.41; integrated–identified 0.89; integrated–introjected 0.11; integrated–external -0.27; integrated–amotivation -0.45; identified–introjected 0.21; identified–external -0.40; identified–amotivation -0.51; introjected–external 0.63; introjected–amotivation 0.56; external–amotivation 0.77. SRMR: REBS 0.093; BSQ-16B 0.057. Median absolute difference between polychoric and ML loadings (REBS) = 0.05; all differences > 0.10 were higher polychoric loadings for skewed items.

Table S11. Exploratory six-factor model of the Romanian REBS (maximum likelihood; fit equivalent to an exploratory structural equation model): standardized loadings after oblique target rotation toward the hypothesized pattern (target loadings in bold; largest cross-loading and its factor shown), and geomin rotation as a check.

Item	Intended factor	Target loading	Largest cross-loading (factor)	Geomin: target loading	Geomin: largest cross-loading (factor)
C1	amotivation	0.18	0.20 (introjected)	0.13	0.22 (introjected)
C2	intrinsic	0.80	0.07 (amotivation)	0.59	0.36 (integrated)
C3	external	0.12	0.40 (introjected)	0.20	0.42 (introjected)
C4	intrinsic	0.96	0.13 (integrated)	0.76	0.20 (integrated)
C5	identified	0.29	0.39 (integrated)	0.26	0.57 (integrated)
C6	amotivation	1.06	0.19 (integrated)	1.00	0.05 (identified)
C7	introjected	0.63	0.17 (amotivation)	0.61	0.20 (identified)
C8	introjected	0.76	0.16 (identified)	0.71	0.23 (identified)
C9	external	0.72	0.13 (introjected)	0.75	0.14 (introjected)
C10	integrated	0.72	0.19 (intrinsic)	0.89	0.03 (identified)
C11	intrinsic	0.32	0.49 (integrated)	0.13	0.71 (integrated)
C12	introjected	0.57	0.27 (integrated)	0.52	0.41 (integrated)
C13	external	0.87	0.10 (integrated)	0.88	0.10 (intrinsic)
C14	amotivation	0.42	0.38 (external)	0.33	0.45 (external)
C15	external	0.80	0.18 (intrinsic)	0.80	0.20 (intrinsic)
C16	identified	0.64	0.22 (integrated)	0.61	0.27 (integrated)
C17	integrated	0.57	0.22 (intrinsic)	0.73	0.15 (identified)
C18	introjected	0.46	0.37 (external)	0.45	0.42 (external)
C19	amotivation	0.14	0.28 (external)	0.07	0.34 (external)
C20	integrated	0.45	0.23 (identified)	0.59	0.20 (identified)
C21	integrated	0.41	0.37 (identified)	0.54	0.33 (identified)
C22	identified	0.87	0.06 (intrinsic)	0.85	0.06 (intrinsic)
C23	identified	0.97	0.03 (introjected)	0.95	0.02 (intrinsic)
C24	intrinsic	0.66	0.14 (integrated)	0.46	0.39 (integrated)

Target-rotated factor correlations: intrinsic–integrated 0.60; intrinsic–identified 0.50; intrinsic–introjected 0.14; intrinsic–external -0.04; intrinsic–amotivation -0.10; integrated–identified 0.51; integrated–introjected 0.02; integrated–external -0.22; integrated–amotivation -0.16; identified–introjected 0.26; identified–external -0.18; identified–amotivation -0.17; introjected–external 0.38; introjected–amotivation 0.29; external–amotivation 0.36.

Items whose largest loading falls on the intended factor: 19/24 (target rotation), 18/24 (geomin). Fit (rotation-invariant): $\chi^2(147) = 226.1$; CFI = 0.974, TLI = 0.951, RMSEA = 0.049, SRMR = 0.025; Satorra–Bentler scaled $\chi^2 = 180.5$ ($c = 1.25$), scaled CFI = 0.985, TLI = 0.972, RMSEA = 0.032 [0.010, 0.047]. ω per factor from the target solution (cross-loadings ignored): intrinsic 0.86, integrated 0.78, identified 0.89, introjected 0.74, external 0.80, amotivation 0.62.

Table S12. BSQ-16B: exploratory one-, two-, and three-factor models, the two-factor geomin solution, and facet-level sensitivity analyses of the substantive models.

Model	χ^2 (df)	CFI	TLI	RMSEA	SRMR	Scaled CFI	Scaled TLI	Scaled RMSEA [90% CI]
1 factor(s)	468.9 (104)	0.872	0.852	0.125	0.060	0.847	0.824	0.112 [0.101, 0.124]
2 factor(s)	213.5 (89)	0.956	0.941	0.079	0.033	0.953	0.937	0.067 [0.053, 0.082]
3 factor(s)	157.7 (75)	0.971	0.954	0.070	0.024	0.969	0.950	0.060 [0.044, 0.076]

Eigenvalues of the item correlation matrix: 9.68, 1.23, 0.80, 0.61, 0.54. Two-factor geomin solution (loadings on facet A = avoidance, body checking, and feeling large; facet B = fear of fatness and preoccupation with shape; factor correlation 0.69):

Item	Facet A	Facet B
B1	0.05	0.84
B2	-0.04	0.94
B3	0.04	0.75
B4	0.38	0.55
B5	0.52	0.21
B6	0.50	0.41
B7	0.51	0.17
B8	0.85	-0.11
B9	0.67	0.24
B10	0.35	0.49
B11	0.52	0.37
B12	0.78	-0.06
B13	0.79	0.10
B14	0.53	0.17

Item	Facet A	Facet B
B15	0.84	-0.09
B16	0.86	0.04

Facet A items: B5, B6, B7, B8, B9, B11, B12, B13, B14, B15, B16; facet B items: B1, B2, B3, B4, B10 (assignment by larger loading); correlation between facet sum scores r = 0.80.

Facet-level models (per SD, HC3-robust 95% CI; covariates as in the primary model). Mirror models with both facets entered together: introjected regulation — facet A β = 0.37 (0.18, 0.55), facet B β = 0.33 (0.14, 0.51); controlled composite — facet A β = 0.36 (0.13, 0.60), facet B β = 0.20 (-0.02, 0.42); autonomous composite — facet A β = -0.06 (-0.32, 0.21), facet B β = 0.06 (-0.18, 0.29). MEDAS model with both facets and the motivational composites: facet A b = 0.59 (0.12, 1.05), facet B b = -0.49 (-0.95, -0.04), autonomous motivation b = 0.64 (0.32, 0.96). Each facet entered alone (with the motivational composites): facet A b = 0.25 (-0.09, 0.60); facet B b = -0.10 (-0.44, 0.23). The opposite-signed coefficients obtained when both facets are entered together reflect a difference-score pattern between two highly correlated, post hoc facets and are not interpreted substantively.

Table S13. Ordinal (WLSMV) confirmatory and exploratory factor models of the Romanian REBS and BSQ-16B estimated in R 4.6.1 with lavaan 0.7-2 (polychoric correlations, diagonally weighted least squares, mean- and variance-adjusted test statistic; N = 224), with the scaled and the robust (Savalei, 2021) fit indices. Script and full output: lavaan_WLSMV.R and lavaan_WLSMV_output.txt in Script S3.

**Model**	**Scaled χ^2 (df)**	**Scaled CFI / TLI**	**Scaled RMSEA [90% CI]**	**Robust CFI / TLI**	**Robust RMSEA [90% CI]**	**SRMR**
REBS						
Six factors, ESEM—ordinal sensitivity analysis [a]	218.2 (147)	0.992 / 0.986	0.047 [0.033–0.059]	0.947 / 0.900	0.090 [0.066–0.113]	0.027
Six correlated factors, independent clusters (hypothesized)	684.6 (237)	0.952 / 0.944	0.092 [0.084–0.100]	0.787 / 0.752	0.142 [0.130–0.155]	0.096
Three factors—rejected	1042.5 (249)	0.915 / 0.906	0.120 [0.112–0.127]	0.712 / 0.680	0.162 [0.150–0.173]	0.130
One factor—rejected	2021.7 (252)	0.811 / 0.793	0.177 [0.170–0.185]	0.482 / 0.433	0.215 [0.205–0.226]	0.203
BSQ-16B						
Bifactor: general + two specific facets—best-fitting	184.3 (88)	0.990 / 0.987	0.070 [0.056–0.084]	0.951 / 0.933	0.096 [0.076–0.116]	0.034
Two correlated facets,	331.1	0.977 /	0.100	0.906 /	0.123	0.050

Model	Scaled χ^2 (df)	Scaled CFI / TLI	Scaled RMSEA [90% CI]	Robust CFI / TLI	Robust RMSEA [90% CI]	SRMR
independent clusters	(103)	0.973	[0.088–0.112]	0.891	[0.106–0.139]	
One factor (hypothesized)—inadequate	468.3 (104)	0.963 / 0.957	0.125 [0.114–0.137]	0.858 / 0.836	0.150 [0.135–0.166]	0.063

Scaled-and-shifted difference tests (Satorra, 2000) against the six-factor independent-clusters model: three factors $\Delta\chi^2(12) = 231.8$, $p < 0.001$; one factor $\Delta\chi^2(15) = 315.7$, $p < 0.001$; exploratory six-factor model $\Delta\chi^2(90) = 175.7$, $p < 0.001$. All models converged; no negative residual variances except in the exploratory six-factor solution (item C6; see below). $p < 0.001$ for all scaled χ^2 tests.

Exploratory six-factor model under WLSMV (target rotation toward the hypothesized pattern): 19 of 24 items had their largest loading on the intended factor, as under maximum likelihood (Table S11); target-rotated standardized loadings are listed in lavaan_WLSMV_output.txt. Factor correlations: intrinsic–integrated 0.66; intrinsic–identified 0.31; integrated–identified 0.32; intrinsic–introjected 0.22; integrated–introjected 0.16; identified–introjected 0.24; intrinsic–external −0.16; integrated–external −0.33; identified–external −0.16; introjected–external 0.27; intrinsic–amotivation −0.26; integrated–amotivation −0.30; identified–amotivation −0.22; introjected–amotivation 0.20; external–amotivation 0.42.

[a] **Note on the robust indices and on item C6.** The robust CFI, TLI, and RMSEA are the categorical-data versions of Savalei (2021) as implemented in lavaan; the scaled versions substitute the mean- and variance-adjusted χ^2 into the maximum-likelihood formulas and are not comparable with the maximum-likelihood reference values (Xia & Yang, 2019). In these data, the robust values are less favorable than the scaled ones for every model; the ranking of the models is the same under both computations, and SRMR, which is comparatively robust to the estimation method (Shi & Maydeu-Olivares, 2020), is unchanged. The robust indices refer to the metric of the continuous responses assumed to underlie the ordered categories, in which the mean absolute polychoric correlations are larger than the corresponding Pearson correlations (0.350 versus 0.266 for the 24 REBS items; 0.638 versus 0.575 for the 16 BSQ-16B items). The WLSMV exploratory six-factor solution is improper: the residual variance of item C6 (amotivation) is −0.32 in the delta parameterization (standardized loading 1.25 on the amotivation factor); the theta parameterization did not converge with target or geomin rotation, and under ULSMV the solution converged with the same item at −0.63 (scaled RMSEA = 0.037, robust RMSEA = 0.100, 90% CI 0.081–0.119; script and output: lavaan_ESEM_diagnostic.R, lavaan_ESEM_diagnostic_output.txt in Script S3). Under maximum likelihood, the uniqueness of C6 reaches its lower bound of zero (target loading 1.06, Table S11): the amotivation factor is weakly determined in the exploratory solution, with C6 showing a boundary loading pattern. Fixing the standardized uniqueness of C6 at 0.05 or 0.10 left the fit of the maximum-likelihood exploratory model unchanged (conventional $\chi^2(148) = 226.4$ and 226.8 versus $\chi^2(147) = 226.1$; Satorra–Bentler scaled $\chi^2 = 181.1$ and 181.4 versus 180.5; scaled CFI = 0.985, TLI = 0.972–0.973, RMSEA = 0.032 [0.010, 0.046–0.047], SRMR = 0.025 in all three cases;

analysis_esem_c6_check.py). The WLSMV exploratory model is reported as an ordinal sensitivity check.

REBS six-factor model, standardized loadings (SE): intrinsic—C2 0.83 (0.02), C4 0.82 (0.02), C11 0.91 (0.03), C24 0.82 (0.03); integrated—C10 0.91 (0.02), C17 0.90 (0.02), C20 0.80 (0.02), C21 0.87 (0.02); identified—C5 0.94 (0.02), C16 0.81 (0.03), C22 0.94 (0.02), C23 0.95 (0.01); introjected—C7 0.69 (0.04), C8 0.66 (0.04), C12 0.63 (0.05), C18 0.83 (0.04); external—C3 0.54 (0.05), C9 0.89 (0.02), C13 0.92 (0.02), C15 0.88 (0.03); amotivation—C1 0.42 (0.06), C6 0.51 (0.06), C14 0.86 (0.04), C19 0.83 (0.04).

Factor correlations: intrinsic–integrated 0.86; intrinsic–identified 0.76; integrated–identified 0.86; introjected–external 0.68; introjected–amotivation 0.60; external–amotivation 0.78; intrinsic–introjected 0.10; integrated–introjected 0.11; identified–introjected 0.22; intrinsic–external −0.22; integrated–external −0.27; identified–external −0.39; intrinsic–amotivation −0.41; integrated–amotivation −0.46; identified–amotivation −0.50.

BSQ-16B one-factor model, standardized loadings (SE): B1 0.85 (0.02), B2 0.86 (0.02), B3 0.75 (0.03), B4 0.86 (0.02), B5 0.72 (0.03), B6 0.87 (0.02), B7 0.69 (0.04), B8 0.82 (0.03), B9 0.89 (0.02), B10 0.79 (0.03), B11 0.83 (0.02), B12 0.85 (0.03), B13 0.87 (0.02), B14 0.66 (0.04), B15 0.77 (0.03), B16 0.88 (0.02).

Table S14. Bifactor model of the BSQ-16B (N = 224): standardized loadings on the general factor and on the specific facet factor under maximum likelihood (Satorra–Bentler scaling) and under WLSMV, item-level explained common variance (I-ECV), and bifactor indices. Facet A (avoidance, body checking, feeling large): B5–B9, B11–B16; facet B (fear of fatness, preoccupation with shape): B1–B4, B10 (from the exploratory two-factor solution, Table S12). General and specific factors are mutually orthogonal.

Item	Facet	General (ML)	Specific (ML)	I-ECV (ML)	General (WLSMV)	Specific (WLSMV)
B1	B	0.74	0.45	0.72	0.77	0.45
B2	B	0.73	0.58	0.62	0.76	0.59
B3	B	0.68	0.36	0.78	0.70	0.38
B4	B	0.82	0.23	0.93	0.85	0.24
B5	A	0.68	0.11	0.98	0.74	−0.01
B6	A	0.89	−0.04	1.00	0.91	−0.08
B7	A	0.64	0.08	0.99	0.72	−0.05
B8	A	0.64	0.50	0.62	0.76	0.41
B9	A	0.83	0.23	0.93	0.88	0.18
B10	B	0.77	0.15	0.96	0.79	0.16
B11	A	0.81	0.08	0.99	0.84	0.08
B12	A	0.62	0.43	0.68	0.81	0.32
B13	A	0.84	0.20	0.95	0.86	0.20
B14	A	0.67	0.08	0.99	0.68	−0.03

Item	Facet	General (ML)	Specific (ML)	I-ECV (ML)	General (WLSMV)	Specific (WLSMV)
B15	A	0.67	0.41	0.73	0.70	0.50
B16	A	0.79	0.40	0.80	0.83	0.41

Fit: $\chi^2(88) = 198.4$; scaled $\chi^2 = 167.7$; scaled CFI = 0.958, TLI = 0.943, RMSEA = 0.064 [0.049–0.078], SRMR = 0.034 (conventional: CFI = 0.961, TLI = 0.947, RMSEA = 0.075). Bifactor indices: ωH = 0.905, ω total = 0.964, ECV = 0.844, PUC = 0.458, H = 0.959; ωHS (specific factors): facet A = 0.080, facet B = 0.168; subscale ω: facet A = 0.946, facet B = 0.922. Comparison models: one factor, $\chi^2(104) = 468.9$ (scaled RMSEA = 0.112); two correlated facets, $\chi^2(103) = 332.5$ (scaled RMSEA = 0.089 [0.076–0.101], CFI = 0.906, SRMR = 0.053; facet correlation 0.86). The models are compared descriptively by their fit and bifactor indices; difference tests are not reported because the one-factor model is a boundary case of both alternatives (a facet correlation of 1 in the two-facet model; zero specific loadings in the bifactor model), where the χ^2 difference test is not standard. The bifactor solution was identical across eight sets of starting values (range of the fit function $< 10^{-9}$, maximum absolute gradient 10^{-6}; analysis_cfa_bifactor.py), and no residual variance was negative. WLSMV solution (lavaan, polychoric metric): general loadings 0.68–0.91, specific loadings −0.08–0.59; ωH = 0.933, ω total = 0.974, ECV = 0.867, H = 0.969; scaled CFI = 0.990, TLI = 0.987, RMSEA = 0.070 [0.056–0.084]; robust CFI = 0.951, TLI = 0.933, RMSEA = 0.096 [0.076–0.116]; SRMR = 0.034; no negative residual variances.

Materials S4. Romanian versions of the instruments as administered (Google Forms), with instructions, response options, and scoring rules. MEDAS items are reproduced verbatim from the questionnaire; see S4.1 and S4.2 for the BSQ-16B and REBS, whose item wordings are available on request, subject to the copyright holders' permission.

S4.0. Study information page (excerpt). Participants were told that the questionnaire was anonymous, that no name, address, e-mail, IP, or other identifying data were collected, that responses would be used exclusively for academic and scientific research purposes, that completion would take about 8–10 minutes, and that submitting the form constituted consent to anonymous processing of the data for the purposes of the study. The questionnaire order was information/consent page, sociodemographic section, BSQ-16B, REBS, MEDAS.

S4.1. Body Shape Questionnaire-16B (BSQ-16B). The BSQ and its short forms are copyrighted (Cooper et al., 1987; Evans & Dolan, 1993) and distributed under license; the Romanian item wordings are therefore not reproduced here and are available from the authors on request, subject to the permission of the copyright holder. As administered, the instruction read: „Am dori să știm cum te-ai simțit în legătură cu aspectul tău în ultimele patru săptămâni. Te rugăm să citești fiecare întrebare și să selectezi numărul corespunzător. Te rugăm să răspunzi la toate întrebările." Response options (shown under every item): 1 = Niciodată; 2 = Rareori; 3 = Uneori; 4 = Des; 5 = Foarte des; 6 = Întotdeauna. Scoring: total = sum of the 16 items (range 16–96); higher scores indicate greater body-shape concern.

S4.2. Regulation of Eating Behaviors Scale (REBS). The REBS (Pelletier et al., 2004) is distributed by the Center for Self-Determination Theory under terms that do not permit republication of the items without the prior written consent of the copyright holder; the

Romanian item wordings are therefore not reproduced here and are available from the authors on request, subject to that permission. The administration instructions, response anchors, and scoring rules as used in this study are given below. Instruction as displayed (in place of the original question stem „Why do you regulate your eating behaviors?”): „Mai jos găsești o serie de afirmații care descriu diferite motive pentru care oamenii încearcă să își regleze comportamentele alimentare (de exemplu, să mănânce mai sănătos, să reducă anumite alimente sau să facă alegeri mai bune). Pentru fiecare afirmație, te rugăm să indici cât de bine reflectă motivul tău personal, folosind scala de mai jos. Selectează un singur număr (1–7) pentru fiecare afirmație, în funcție de cât de mult te regăsești în ea.” Response scale 1–7 with labeled anchors (shown under every item): 1 = nu corespunde deloc motivelor mele (această afirmație nu mă reprezintă, nu este motivul pentru care aș încerca să-mi reglez alimentația); 4 = corespunde moderat motivelor mele (parțial adevărat pentru mine); 7 = corespunde exact motivelor mele (această afirmație mă descrie perfect, este exact motivul meu). Scoring (item means): intrinsic C2, C4, C11, C24; integrated C10, C17, C20, C21; identified C5, C16, C22, C23; introjected C7, C8, C12, C18; external C3, C9, C13, C15; amotivation C1, C6, C14, C19. Autonomous composite = mean of the 12 intrinsic, integrated, and identified items; controlled composite = mean of the 8 introjected and external items.

S4.3. Romanian Adaptation of the 14-item Mediterranean Diet Adherence Screener (MEDAS; PREDIMED criteria). The MEDAS items were published in open-access articles (Schröder et al., 2011; Martínez-González et al., 2012, the latter under a Creative Commons Attribution license) and are reproduced here in Romanian adaptation with attribution to the original authors. Instruction as displayed: „Răspunde la următoarele întrebări despre obiceiurile tale alimentare.” Response options: DA / NU. Scoring: each „DA” scores 1 point; total 0–14; low 0–5, moderate 6–9, high ≥ 10. These are the conventional cut points of the original MEDAS; they were applied descriptively and in the ordinal sensitivity analysis but have not been independently validated for this Romanian-adapted implementation. The 13-item sensitivity score omits item D8 (wine). Frequency thresholds were retained from the original instrument, but several original gram-based serving definitions and the requirement that at least one daily vegetable serving be raw or as salad were not displayed; the wording below is reproduced exactly as administered. Local food examples were added where indicated.

D1. Folosești ulei de măsline ca sursă principală de grăsime pentru gătit?
D2. Consumi 4 linguri (sau mai mult) de ulei de măsline pe zi (inclusiv uleiul folosit la gătit, salate sau în mâncăruri consumate în afara casei)?
D3. Consumi 2 sau mai multe porții de legume pe zi? (1 porție = ≥200 g crud sau ≥150 g gătit)
D4. Consumi 3 sau mai multe porții de fructe pe zi? (Include fructe întregi, sucuri și porții separate consumate în orice moment al zilei.)
D5. Consumi mai puțin de 1 porție de carne roșie, hamburgeri sau carne procesată pe zi? (Carne procesată = salam, crenvurști, cârnați, bacon, șuncă, mezeluri etc.)
D6. Consumi mai puțin de 1 porție de unt, margarină sau smântână pe zi?
D7. Consumi mai puțin de 1 băutură îndulcită și/sau carbogazoasă pe zi? (Băuturi răcoritoare, băuturi carbogazoase, ceaiuri îndulcite, sucuri industriale.)
D8. Bei vin? (7 sau mai multe pahare pe săptămână) (≈ 1 pahar pe zi; 1 pahar = 100–150 ml)
D9. Consumi leguminoase ≥3 ori/săptămână? (Fasole, linte, năut, mazăre.)
D10. Consumi pește sau fructe de mare de 3 sau mai multe ori pe săptămână?
D11. Consumi produse de patiserie comerciale (nu făcute acasă) de mai puțin de 3 ori pe

săptămână?
D12. Consumi nuci de 3 sau mai multe ori pe săptămână?
D13. Consumi preparate gătite cu sos de roșii, usturoi, ceapă sau praz în ulei de măsline (sofrito) de 2 sau mai multe ori pe săptămână?
D14. Consumi carne albă (pui, curcan) mai des decât carne roșie?

Data S1. De-identified item-level dataset (Supplementary_Data_S1_minimal_dataset.xlsx: worksheets Data, Codebook, README; N = 225 rows, exact duplicate flagged with inclus_analiza = 0, the four comprehensibility-pretest respondents flagged with pretest = 1) and SPSS scoring syntax (Supplementary_Data_S1_SPSS_syntax.sps). Relative to the minimal dataset provided with the original submission, the variables current_style, weight_mod, and emo_eating were added in the first revision, and the flag variable pretest in the second revision (Table 5, pretest-exclusion sensitivity analysis); all other columns are unchanged.
